# High-Throughput Preparation of Uncontaminated Graphene-Oxide Aqueous Dispersions with Antioxidant Properties by Semi-Automated Diffusion Dialysis

**DOI:** 10.3390/nano12234159

**Published:** 2022-11-24

**Authors:** Ivan V. Mikheev, Sofiya M. Byvsheva, Madina M. Sozarukova, Sergey Yu. Kottsov, Elena V. Proskurnina, Mikhail A. Proskurnin

**Affiliations:** 1 Department of Chemistry, Lomonosov Moscow State University, Moscow 119991, Russia; 2 Kurnakov Institute of General and Inorganic Chemistry, Russian Academy of Sciences, Moscow 117901, Russia; 3 Research Centre for Medical Genetics, Moscow 115522, Russia

**Keywords:** purification, graphene oxide, dialysis, chemiluminescence, free-radical regulation, radical scavengers

## Abstract

A semi-automated diffusion-dialysis purification procedure is proposed for the preparation of uncontaminated graphene oxide (GO) aqueous dispersions. The purification process is integrated with analytical-signal processing to control the purification degree online by several channels: oxidation-reduction potential, conductivity, and absorbance. This approach reduces the amounts of reagents for chemical treatment during dialysis. The total transition metal (Mn and Ti) content was reduced to a sub-ppb level (assessed by slurry nebulization in inductively coupled plasma optical atomic emission spectroscopy). Purified aqueous GO samples possess good stability for about a year with a zeta-potential of ca. −40 mV and a lateral size of ca. sub-µm. Purified GO samples showed increased antioxidant properties (up to five times compared to initial samples according to chemiluminometry by superoxide-radical (O_2_^−^) generated in situ from xanthine and xanthine oxidase with the lucigenin probe) and significantly decreased peroxidase-like activity (assessed by the H_2_O_2_–L-012 system).

## 1. Introduction

Graphene oxide (GO) is a two-dimensional material with a unique atomic structure, tunable lateral size, high specific surface area, and variety of *sp*^3^ carbon atoms with functional oxygen-containing groups [1]. The reduction of GO to reduced graphene oxide (rGO) gives new perspectives for tunable preparation of graphene-resemblance structures [2]. The unique properties of both GO and rGO pave the way for a wide range of applications in technology, biomedicine, and environmental sciences [3,4]. Robust membranes based on GO or rGO can be prepared for desalination, separation of gaseous mixtures, and water purification [5]. In biomedical applications, GO, especially aqueous GO dispersions (aqGO) can be used as a tunable material for wearable artificial kidneys [6,7] or thin-film nanofiltration purification systems [8]. GO and rGO have a potential in diagnosis and treatment of cancer [9,10,11], drug delivery [12], in tissue regeneration and immunomodulation [13,14]. Studies of nanozyme properties of GO and rGO are being actively performed [15,16].

Many applications require pure GO-based materials, especially for biomedical and other practical uses, e.g., heat-transfer tasks or optical materials; thus, purification steps are on the cutting edge of many applications of GO and rGO [17]. E.g., graphene nanoplatelets bridge between two semiconductors (AgBr and graphitic carbon nitride) boost photoelectrochemical performance [18]. Effective photocatalysts generated the superoxide radical (O_2_^−^) and holes (h^+^) were used to achieve the complete degradation of tetracycline hydrochloride [19]. Graphene oxide-TiO_2_ composite was used for the degradation and mineralization of two hazardous pollutants—diphenhydramine pharmaceutical and methyl orange azo dye—under both near-UV/Vis and visible light irradiation [20].

There are several preparation procedures of GO [21] and rGO [22], but chemical exfoliation demands a strong oxidant which remains as a contaminant. The main purification issues of GO involve removing Mn^7+^ as a primary oxidizing agent, KMnO_4_ and in-situ forming Mn^4+^/Mn^2+^ species [23] as well as other transition metals [24]. Tunable reduction of Mn^4+^ to Mn^2+^ by oxalic acid effectively removes Mn species [25]. Secondly, purification from sulfur as anion contaminants is of importance [25,26,27]. Thirdly, the purification target is oxidative debris [28], also referred to as carbonaceous garbage [29] that consists of oxidized polyaromatic fragments adsorbed on GO nanoplatelets [30]. They should be removed due to their negative impact on the effective surface area [31].

Despite numerous studies, as a whole, the issues of green and sustainable preparation of pure GO and rGO materials are still open [32] as almost all techniques of purifying GO from impurities appear to be not fully developed. There are two main approaches for purification of GO samples. The first, ‘washing’ approach involves aqueous reagents (mainly strong acids like HCl) and centrifugation for rapid separation of the washed GO. Methods for the preparation of ultra-pure salt-washed graphene oxide (swGO) have been proposed [33]; however, the authors note the need for further development of purification methods.

Contrary to washing, another, deeper and prolonged, GO purification approach is based on diffusion dialysis (DD). Numerous studies have shown the capability of dialysis bags to purify graphene-based nanomaterials rapidly; scaling using a vacuum dialysis system was studied, allowing up to 400 g GO to be purified within two days [34]. Dialysis purification is integral to the large-scale preparation of oxidized graphene [2]. The automated DD increases the efficiency of purification, though there is no data on such an approach for GO materials.

DD approaches result in highly pure GO samples with special properties. Highly purified GO may possess free-radical scavenging activity by the main sites related to honeycomb lattice structures by *sp*^2^-hybridized carbon atoms [35], which act through adducts or electron transfer formation [36]. On the contrary, the purification of graphene oxide or its synthesis under specific conditions leads to a decrease in the electrocatalytic activity with respect to the oxygen reduction reaction [37]. We assume that many accessible functional groups cause the high activity of graphene due to purification: (1) the network of conjugated flatted double bonds and (2) surface moieties that can contribute to catalytic activity. However, there is still no evidence of estimation of such properties and comparison for purified and non-purified GO samples.

The metabolism of reactive oxygen species (ROS) is basic for living organisms. Primary ROS include the superoxide radical anion and hydrogen peroxide. The toxicity of nanopreparations to cells or the cytoprotective effect is largely determined by their relationship to reactive oxygen species and the ability to exhibit nanozyme oxidative properties. These are properties of mimetics of superoxide dismutase, catalase, and peroxidase. There are several studies in which oxidative properties of graphene oxide and the cytotoxicity of graphene associated with its prooxidative properties is reported [38,39,40]. Graphene oxide is reported to induce lipid peroxidation [41]. Oxidative debris present in as-prepared graphene oxide plays a very important role in the pathogenesis of this toxicity [42]. Moreover, trace impurities of metals of variable valence (iron, copper, and manganese) in graphene may show their own high activity in relation to ROS [43,44]. Therefore, when studying the oxidative properties of graphene and its derivatives, one must be sure of the purity of the preparation. Thus, models of free radical biochemical reactions can be used, on the one hand, as proof of the importance of deep purification of graphene. On the other hand, studies on high-purity graphene will provide new knowledge about its true biochemical activity.

Thus, this work aims to develop a sustainable, automated, and green approach (setup and procedure) for aqGO purification by reagent supply regulation (reducing quantities and minimizing wastewater runoff) and to evaluate oxidative nanozyme properties of highly purified aqGO samples compared to commercially available non-purified ones using biochemical model systems.

## 2. Materials and Methods

### 2.1. Materials

Graphene oxide (GO) with 1.25 C:O mole ratio and reduced graphene oxide (rGO) (LLC Rusgraphene, Moscow, Russia) and monolayer content more than 80% was used throughout.

#### Bulk GO Samples Characterization

Purity of bulk GO and rGO samples was estimated by direct slurry nebulization ICP-AES analysis [45]. For GO (in *w*/*w* % Mn < 0.50, K < 0.25, S 2.0 ± 0.2); the C:O ratio was 1.5 by gravimetry, sample moisture, <0.02 mass %; for rGO (in *w*/*w* % Mn < 0.50, K < 0.25, S 0.25 ± 0.04); the C:O ratio was 1.96 on average by gravimetry and XPS (see Supplementary Materials, Sections S1.2 and S1.3, Table S3 and Figure S6, respectively), sample moisture, <0.02 mass %.

### 2.2. Reagents

All reagents were chemically pure or pure grade which were used as received without preliminary purification. Reagents for automation dialysis were: (1) Hydrogen peroxide (stabilized by H_3_PO_4_, pure assay (titr.): min. 37%), (Peroxide Ltd., Moscow, Russia); (2) ethylenediaminetetraacetic acid (EDTA), ACS reagent grade, 98% (Merck, Darmstadt, Germany); (3) ultrapure water Milli-Q^®^ 18.2 MΩ·cm at 25 °C with TOC-level less 3 ppb (Millipore Corp., Darmstadt, Germany). Potassium chloride 99.999% assay was used for the technical maintenance of reference (Ag|AgCl) and working (pH) electrodes.

The 100 mM phosphate buffer solutions (PBS) with pH 7.4 and 8.6 were prepared by dissolving KH_2_PO_4_ (Sigma-Aldrich, St. Louis, MO, USA) in 1.00 L of ultrapure water followed by the adjustment to the desired pH value using granular potassium hydroxide (Sigma-Aldrich, St. Louis, MO, USA).

#### 2.2.1. Enzymes, Substrates, and Chemiluminescent Probes

Catalase from bovine liver (Cat), lyophilized powder (2000–5000 units/mg protein) and peroxidase (Px) from horse radish, 10,000 U/vial with working concentrations of 7 mM and 200 nM, respectively, were prepared by dissolving in a PBS with pH 7.4 (Sigma-Aldrich, St. Louis, MO, USA). A xanthine oxidase (XO) working solution (Sigma-Aldrich, St. Louis, MO, USA) with an activity of 0.11 U/mL was obtained by diluting the PBS suspension (pH 7.4). Before chemiluminescence measurements, the stock enzyme solution was kept for 15 min at laboratory temperature. The catalytic activity of the one enzyme unit converts 1.0 μmol of xanthine to uric acid in 1 min at 25 °C and pH 7.5.

Chemiluminescent probes L-012 (8-Amino-5-chloro-2,3-dihydro-7-phenyl-Pyrido [3,4-d]pyridazine-1,4-dione, sodium salt, 8-Amino-5-chloro-7-phenyl-2,3-dihydro-pyrido [3,4-d]pyridazine-1,4-dione, sodium salt) and Luminol (3-Aminophthalhydrazide, 5-Amino-2,3-dihydro-1,4-phthalazinedione; Sigma-Aldrich, St. Louis, MO, USA) were used for the assessment of peroxidase/catalase activity by chemiluminescence. Its stock solution of 1 mM was prepared by dissolving a sample in PBS (pH 7.4). A selective superoxide anion-radical of a chemiluminescent probe, lucigenin (10-methyl-9-(10-methylacridin-10-ium-9-yl)acridin-10-ium dinitrate (Sigma-Aldrich, St. Louis, MO, USA), with working concentrations of 1 mM, was prepared by dissolving in PBS with pH 8.6.

A xanthine (Xa), 3,7-dihydropurine-2,6-dione (Sigma-Aldrich, St. Louis, MO, USA) aqueous solution was prepared. An off-the-shelf ultrasound probe with a MEF93.T timer at continuous operation and 0.6 kW electrical-power mode was used for GO aqueous solution preparation (LLC MELFIZ-ul’trazvuk, Moscow, Russia). Parameters of the ultrasonic device and the operating mode are described elsewhere [46]. A Thermo Fisher Scientific FBH 612 low-temperature circulation thermostat (Thermo Fisher, Waltham, MA, USA) was used for increased ultrasound efficiency during aqGO preparation.

#### 2.2.2. Certified Reference Materials (CRMs)

Elemental analysis. ICP-AM-6-A ICP Analytical Mixture with 23 components solution for instrument calibration solutions and elemental analysis on ICP-OES (High Purity Standards, North Charleston, SC, USA) was used throughout.

Electrochemical measurements. A 1413 µS/cm conductivity standard solution was used to calibrate the conductometer according to NIST recommendations [47]; pH-meter calibration was performed using NIST Traceable standard buffer solutions with pHs of 1.68, 4.01, 6.68, 9.18, and 11.00 (Hanna Instruments, Woonsocket, RI, USA) according to IUPAC recommendations [48].

Inorganic anion analysis. Multielement ion chromatography certified standard solution 10 mg/L of F^−^, Cl^−^, NO_3_^−^, PO_4_^3−^, and SO_4_^2−^ used for ion chromatography calibration (ROTI^®^Star Fluka Analytical, Buchs, Switzerland).

### 2.3. Dialysis Setup Equipment and Operation

We used off-the-shelf cellulose membrane tubing with a molecular weight cutoff (MWCO) at 3.5 and 14 kDa Spectra/Por™ 3 Spectrum Labs (Thermo Scientific, Waltham, MA, USA). Pre-conditioning of freshly packed tubes was performed by procedure 2 below to wash out glycerol, sulfide, and trace transition metals.

Dialysis setup was equipped with (1) a magnetic stirrer with a hot plate (ISO Lab, Isolab Laborgeräte GmbH, Eschau, Germany), (2) a 4-channel auto dosing peristaltic pump, Jebao Doser 3.4 WiFi programmable (Zhongshan Jiebao Electronic Appliance Co., Ltd., Zhongshan, China), (3) a washing solution channel compartment including three 10 L polypropylene tanks with aqueous solutions of 0.005 M EDTA, 3 wt.% H_2_O_2_, ultrapure water, and drain (see the Supplementary Materials, Figure S1). This setup requires maintenance every 500 h of peristaltic pump operation: replacement of feed tubes and the lubricate pump shaft to sustain reproducible dosing results.

Analytical sensors for precise online measurement of oxidation-reduction potential (ORP), conductivity, and absorbance were used: (1) an Expert-001-2 ORP meter (Econix-Expert, Ltd., Moscow, Russia) equipped with a double-junction working ORP Pt-electrode—MA921B/2 (Milwaukee Instruments, Melrose, MA, USA) and an Ag|AgCl reference electrode (Expert, Ltd., Moscow, Russia); (2) an Expert-002 electrical conductivity meter (EC meter, Econix-Expert, Ltd., Moscow, Russia); and (3) a Cary^®^ 50 UV-Vis Spectrophotometer with a fiber optic coupler was operated at 190–1100 nm for absorbance measurements (Agilent, Mulgrave, Australia). An IPPON Innova G2 1000 (Ippon Ltd., Hong Kong, China) uninterrupted power supply (UPS) with a double conversion of electrical power was used to conduct uninterrupted analytical signal processing.

### 2.4. Other Instrumentation

A SevenCompact™ pH/Ion S220 pH-meter (Mettler-Toledo AG, Greifensee, Switzerland) was used to prepare the phosphate buffer solution. An axial view Agilent 720 ICP-OES spectrometer (Agilent, Mulgrav, Australia) was used for elemental analysis. The ICP-AES operating conditions are given elsewhere [46]. Atomic emission lines are chosen based on ISO 11885:2007 recommendations.

An Agilent Cary 4000 spectrophotometer (Agilent, Mulgrave, Australia) was used for GO’s UV/Vis spectra recording. An ISC-1600 ion chromatograph (Thermo Fisher Scientific, Oxford, UK) equipped with a particular chromatographic column Dionex IonPac (Thermo Fisher Scientific, Sunnyvale, CA, USA) AS4 (4 mm I.D., length 250 mm) with a precolumn Dionex IonPac AG4 (4 mm I.D., length 50 mm) was used for anion analysis ($F^{-},{\mathrm{Cl}}^{-},{\mathrm{NO}}_{3}^{-},{\mathrm{SO}}_{4}^{2-},{\;\mathrm{PO}}_{4}^{3-}$). Before the analysis, all GO samples were centrifugated by Armed 80-2s (LLC Armed, Moscow, Russia). The colloidal properties of aqueous GO particles (size distribution and zeta-potential) were assessed by a ZetaSizer Nano ZS (Malvern Instruments, Malvern, UK) dynamic light scattering (DLS) instrument, operating at 25 °C; the angle of backscattering, 173°. Total organic carbon analysis was conducted by a TOC-L carbon analyzer (Shimadzu, Kyoto, Japan). ATR-FTIR spectra for GO functional-group analysis were recorded on a Bruker Vertex 70 single-beam IR Fourier spectrometer (Bruker Optik GmbH, Ettlingen, Germany) equipped with a GladiATR™ (PIKE Technologies, Fitchburg, WI, USA) monolithic diamond ATR for full spectral range from 4000–400 cm^−1^ and into the far-IR, which is described elsewhere [49].

Nanozyme oxidative activity (SOD-like, peroxidase-like, and catalase-like) was studied with an enhanced chemiluminescence method. Chemiluminescence measurements were carried out with a Lum-1200 12-channel chemiluminometer (DISoft, Moscow, Russia). The chemiluminometer detects visible light in a range of 300–700 nm; no bandpass filters were used. Signal processing was performed via PowerGraph v.3.3.11 Professional software (DISoft, Moscow, Russia) [50].

### 2.5. Data Treatment

The measurement results are presented as mean values with confidence intervals (*p* < 0.05 or *p* = 0.95) under the requirements for the competence of testing and calibration laboratories ISO 17025:2020. Calibration parameters were calculated according to the IUPAC recommendations to present the results of the chemical analysis [51]. All statistical analysis and plot illustration were conducted by Origin 2017 SR1 b9.2.257 (OriginLab Corporation, Northampton, MA, USA).

### 2.6. Procedures and Protocols

**Procedure 1. Preparation of GO aqueous dispersions**

A weighted portion of GO (or rGO) samples ca. 50 ÷ 150 mg was placed into a 100 mL Erlenmeyer flask. A 75-mL portion of pure water was put into a flask, then ultrasound probe working in a continuous mode was immersed. Then, the flask was placed into a thermostat at 25 °C. The ultrasound graphene oxide exfoliation was conducted four times for 30 min with 15 min breaks between cycles. Then, ultrasound-exfoliated graphene oxide was placed into a 100.0 mL volumetric flask, and water was added to the mark.

**Procedure 2. Pre-conditioning membrane (dialysis bag) treatment for dialytic purification of graphene oxide**

The necessary length of the membrane (dialysis bag) for the treatment according to the manufacturer recommendations about size vs. volume was cut. A portion of 150 mL of 5 mM EDTA put into a conical (Erlenmeyer) flask. The membrane was placed into the flask and the surface was thoroughly wetted. The flask was heated to 60 °C under stirring and left for three days. The chemicals were exchanged three times every 24 h. After the preconditioning, the membrane was flushed using 200 mL of ultrapure water.

**Procedure 3. Semi-automatic dialysis purification of graphite oxide aqueous dispersions using EDTA and hydrogen peroxide**

**Figure 1:**
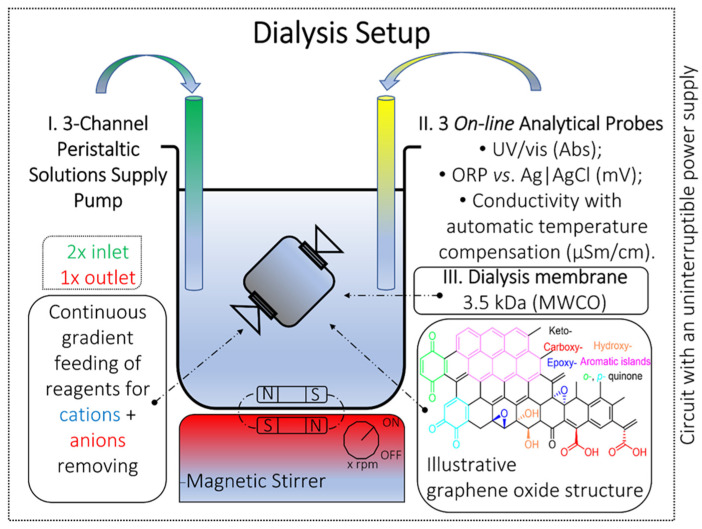
Schematic diagram of the dialysis setup.

A portion of 10 mL of aqGO was placed into a pre-conditioned membrane (procedure 2), covered with a polypropylene clamp, and put into a 1 L chemical beaker of ca. 800 mL of deionized water. Automatic supplies of reagents, washing liquid, and drainage were connected to the system using three channels of the peristaltic pump (see the Supplementary Materials, Figure S1). Automated cyclic feeding reagents of 100 mL per cycle were performed in a programmable mode every 30 min (see the Supplementary Materials, Table S1). During the first four days, the dialysis was performed in a reagent-supply mode in the following channels: (1) 0.05 M EDTA, (2) 3% *w*/*v* hydrogen peroxide, and (3) drain. During dialysis, the subsequent analytical signals were monitored: (1) electric conductivity (μS/cm), ORP (mV), and absorption spectrum (a.u.) of the washing liquid in the range 190–1100 nm. The setup schematic is shown in Figure 1. After dialysis, the sample was transferred into a brown borosilicate-glass vial, and the dialysis purification efficiency was evaluated using ICP-AES, TOC, and FTIR.

**Procedure 4. Chemiluminometric assay of superoxide dismutase-like activity**

Superoxide anion-radical (SAR) scavenging potential of aqGO was assessed by chemiluminometry with the xanthine and xanthine oxidase (Xa/XO) model system in the presence of the lucigenin probe as a selective enhancer for SAR, which we presented for fullerenes [52]. Briefly, aliquots of xanthine (20 μL, 20 μM), lucigenin (20 μL, 1 μM), and the analyzed GO samples were added to a cuvette with PBS (100 mM, pH 7.4). The background signals of luminescence were recorded for 30 ÷ 60 s, then an aliquot of xanthine oxidase (40 μL; activity, 4.4 mU/mL) was added. The total volume of the system was 1.00 mL. All samples were analyzed in triplicate at 37 °C, and signal registration was processed up to 15 ÷ 20 min. As the analytical signal, the ratio of areas under the curve to for the test and blank experiments (without GO) was used.

**Procedure 5. Chemiluminometric assay of peroxidase- and catalase-like activities**

The peroxidase- or catalase-like activity of the analyzed GO samples was studied in the luminol (or a chemiluminescence probe L-012) and H_2_O_2_ system. A solution of luminol or L-012 (40 µL, 1 mM) was prepared by dissolving a sample in PBS (pH 7.4 and 8.6). A working solution of hydrogen peroxide with a concentration of 0.1 mM was prepared by diluting the 37 *w*/*v* % H_2_O_2_ stock solution with ultrapure water. Background signals of luminescence were recorded for 30 ÷ 60 s, then an aliquot of H_2_O_2_ (10 µL, 0.1 mM) was added, then, in another 30 s, an aliquot of peroxidase (20 μL, 20 nM) or catalase (10 μL, 7 µM), finally, in another 60 ÷ 120 s, an aliquot of GO (10 μL, ca. 1 g/L). The total volume of the system was 1.00 mL. All samples were analyzed in triplicate at 37 °C, and signal registration was up to 15 ÷ 20 min. As the analytical signal, the ratio of areas under the curve to for the test and blank experiments (without GO) was used.

## 3. Results

### 3.1. Dialysis Dispersion Purification and Monitoring

GO aqueous dispersions were prepared by Procedure 1 using ultrasound-assisted exfoliation [53] and GO powder. The total time of all the stages of ultrasound preparation of dispersions is 3.5 h. We used a thermostat to sustain no attenuation of ultrasound power in solution [54], increase the reproducibility of the preparation yield, and reduce evaporation. Due to light irradiation effects on the decomposition of graphene oxide [55], the storage was in the dark.

After preparation, GO dispersion was purified. The purification strategy can be summarized as: (i) the removal of any contaminants by selecting a ready-to-use target reagent for a two-step treatment based on preliminary sample characterization and impurity affinities; and (ii) analytical control and monitoring: handling and controlling the reagent supply; monitoring specific parameters to help spotting a purification problem at any stage; and the precise quality control of the final product. Dialysis purification includes several steps (Figure 1): (1) dialysis bag preconditioning (procedure 2); (2) dialysis purification with multiparametric monitoring and end-point dialysis estimation (procedure 3); and (3) pure aqGO characterization.

**Figure 2:**
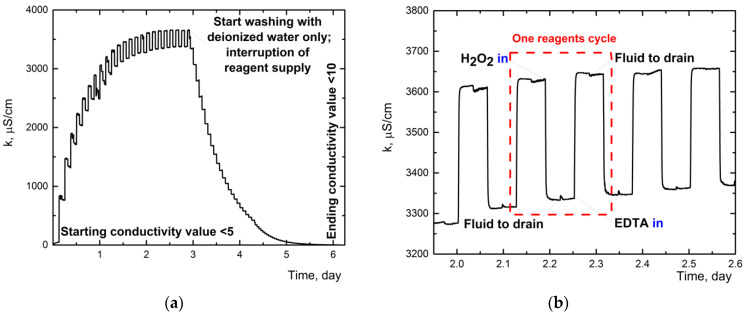
The profile of conductivity signal (κ) during the dialysis process: (**a**) overall process with the wash-out step with ultrapure water; the endpoint corresponds to the electrical conductivity lower than 10 µS/cm, signal logging every 1 s for 6 days. (**b**) Zoomed area for the plot (**a**) for the value of conductivity when filling the purification reagents (non-electrolyte H_2_O_2_ decrease of κ and electrolyte EDTA increase of κ) between 2.5 and 3 days.

**Figure 3:**
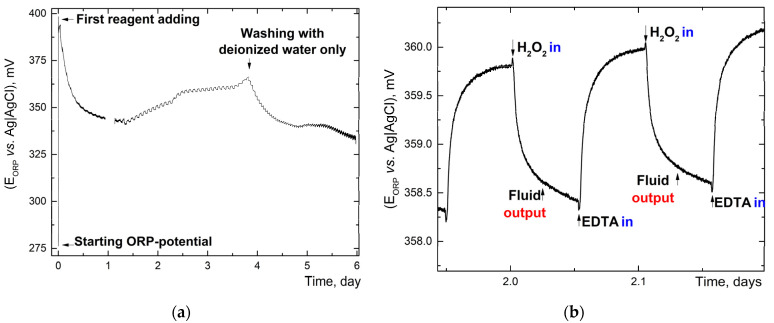
The profile of ORP potentials during the dialysis process. (**a**) Overall process. (**b**) Increased scale of ORP potential when filling the purification reagents (H_2_O_2_ and EDTA). EDTA increases ionic strength of the solution and changes ORP. Signal logging every 1 s for 6 days.

**Figure 4:**
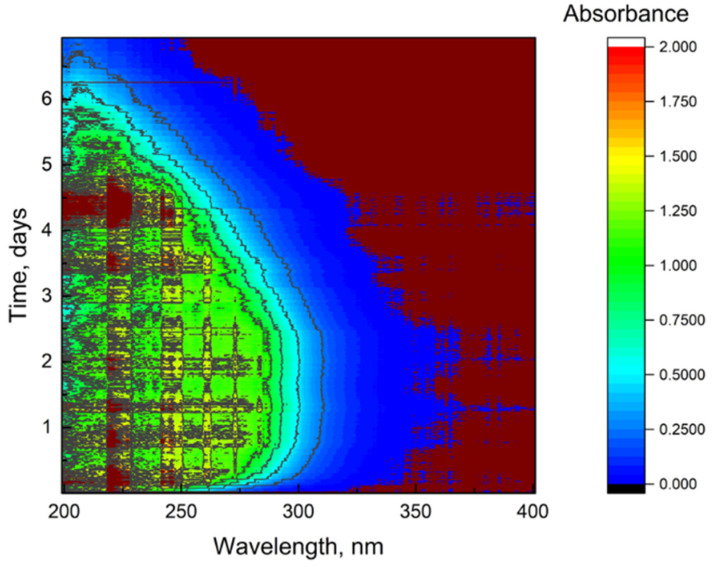
The profile of 1000 collected absorbance spectra from a fiber-optic probe of dialytic solution during the process, range 190–400 nm. The scanning pitch is 1 nm. The scan rate is 60 nm/min.

During the dialysis stage, the feeding of reagents for purification was carried out in an automatic mode. Reagent containers were pre-soaked with target reagents for 30 days (conditions are approximately the same as [56]) to remove plasticizers and unrelated impurities. The results of plasticizer quantities (phthalates) after 60-day storage are presented, which do not exceed 0.05 ppm in some cases (Supplementary Materials, Section S1.4, Figures S7–S10, Table S4). The following analytical signals from probes were also monitored during the process: conductivity (Figure 2), ORP (Figure 3), and absorbance spectra (Figure 4). The quality control procedure depends on the type of contaminants, transition metals used as oxidation products, and extra exfoliation procedure.

The optimum conditions to prepare highly pure GO dispersions are summed up in Table 1. Using a 0.05 M EDTA and a 3% *w*/*v* hydrogen peroxide as reagents, followed by washing with distilled water for 3 days, allows purifying GO samples from manganese and aluminum thoroughly and reduces the titanium (by 90%) and iron (by 80%) to the ppb level. Using membranes with a 14 kDa molecular weight cutoff (MWCO) is optimal.

More specific comments are presented in Table 1. Excess purification reagents (EDTA and H_2_O_2_) were removed by dialysis by an ultrapure water supply after 3 days following the reagent-supply stage. Below we describe each step in more detail.

#### 3.1.1. Monitoring Metal Contents in Membrane during Preconditioning

Due to the high content of metals [57] and sulfides [58], there is a need of preliminary dialysis bag preconditioning (procedure 2). Preconditioning dialysis membranes with a 0.05 M EDTA solution reduced the degree of contamination of GO dispersions with transition metals. An untreated membrane increases copper and zinc contents up to 400%.

#### 3.1.2. Conductivity Monitoring

Electrical conductivity (EC) was used as the primary channel, and the dialysis was stopped when this parameter reached the threshold of 10 µS/cm (Figure 2a). EC measurement in dialysate solution shows a behavior similar to ORP, see below. The beginning of signal registration started at values of less than 5 μS/cm. The overall profile is shown in Figure 2a, there was a gradual increase in EC to the steady state. Adding uncharged hydrogen peroxide lowers EC (dilution), charged EDTA increases the EC value (Figure 2b).

#### 3.1.3. Oxidation-Reduction Potential Monitoring

ORP has a sharp increase at the beginning of reagent feeding, then, for one day, it dropped to the steady-state value (Figure 3a). The increase is associated with launching catalytic decomposition of hydrogen peroxide by manganese dioxide (which is a component of crude graphene oxide). Long return to steady-state values is most likely due to adsorption of oxidation products on the platinum electrode.

Between one and four days, it tends to have a gradual increase. A detailed examination of the signal shows up-and-down behavior due to the supply of purification reagents (Figure 3b). Adding uncharged hydrogen peroxide lowers ORP by dilution; adding a charged EDTA, on the contrary, increases the potential as EDTA increased the solution ionic strength. There was a rapid decrease in the final washing-out phase. As well, a slight increase in ORP after adding each portion of water is observed (Figure 3a). On the fifth day, the potential remained stable, indirectly indicating the completion of the main purification stage (Figure 3a). After five days, the relative standard deviation of ORP measurements increases (the signal-to-noise ratio decreases) due to the insufficient ionic strength of the solution [59]. A similar behavior was observed for a 14-day experiment.

#### 3.1.4. UV/vis Absorbance Spectra with Fiber Optical-Probe Monitoring

A contour map of absorption spectra during the purification is shown in Figure 4 (ca. 1000 spectrums). First, one can see an increase in absorption due to EDTA feeding. The absorption in the range of 190–300 nm remains unchanged owing to a constant concentration of this substrate. It is also worth noting that there is a minor contribution from graphite oxide, which also passes through the dialysis membrane. After adding water for washing, the absorption decreases. In the end of the process, the significant absorption is observed only in the range of 190–220 nm. As well, 254 nm has been selected for carbonaceous content release during dialysis (see the Supplementary Materials, Figure S2A).

### 3.2. Properties of Purified Graphene Oxide Dispersions

#### 3.2.1. Total Yield

The total organic carbon (TOC) content estimation after preparation of aqGO (Procedure 1) made it possible to check that the share of the non-dispersed and unstable GO fraction was less than 5%. Due to the high lateral size of GO, filtering through 0.45 µm removed almost all its fraction (Table 2). Total losses of the carbon fraction were up to 70%. A low TOC loss is shown for the 3.5 kDa membrane to be approx. 25%. Reusing a 3.5 kDa membrane decreased TOC up to 2.5 times. For 14 kDa membranes, the maximum TOC loss was approx. 50%. Reusing a 14 kDa membrane decreased TOC up to 1.05 times. On average, the losses for a 14 kDa membrane were 1.6 times higher than for a 3.5 kDa membrane. In terms of dialysis bag stability, we evaluated the change and shift in IR absorption spectra by ATR-FTIR, which coincided with all presented bands before and after the dialysis in the range 4000–100 cm^−1^ (Figure S3A,B in the Supplementary Materials). No additional bands are observed in FTIR spectra, not even graphene oxide bands, which were visually retained in the membrane.

#### 3.2.2. Sample Stability Lateral Size and Zeta-Potential

After purification, we estimated the sedimentation stability by UV/vis spectra and zeta-potential measurements (Table 2). The wavelength of 254 nm was selected as characteristic for the GO samples in analogy with dissolved organic carbon [60]. In all cases, absorbance decreased to 10% for 8 months, showing excellent stability. In all cases, the absorbance of purified samples at 254 nm decreased no more than 10% for 8 months, showing excellent stability after purification.

#### 3.2.3. pH and Conductivity

The acidity of GO samples increases after purification, reflecting a decrease in sulfate from sulfuric acid used for the graphite exfoliation process (Table 2). Measurements of anions conducted by ion chromatography after 5 min centrifugation at 4 krpm showed that, with both 3 and 14 kDa membranes, sulfate and nitrate contents decreased up to 97%. These data correlate with conductivity measurements (decreased on average to 9 times).

#### 3.2.4. UV/Vis Absorption Spectra

All absorption bands registered in the UV/vis region (Supplementary Materials, Figure S2B) coincide with the previously known for the UV/vis region [61]. The plasmon peak at 210 nm belongs to π→π* of C=C bonds and 310 nm, to n→π* transitions due to organic peroxide (R–O–O–R) or epoxide (C–O–C) moieties. The weak bands at 600 and 730 nm correspond to GO in solution [61]. After dialysis, there is no band shifting; however, increasing absorbance in normalized absorption spectra is revealed.

#### 3.2.5. FTIR Absorption Spectra

**Figure 5:**
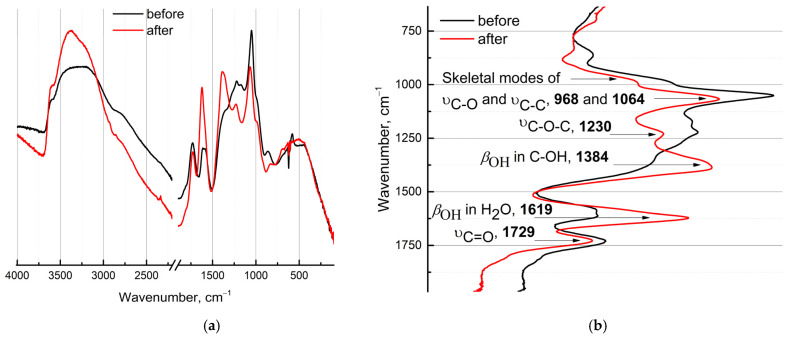

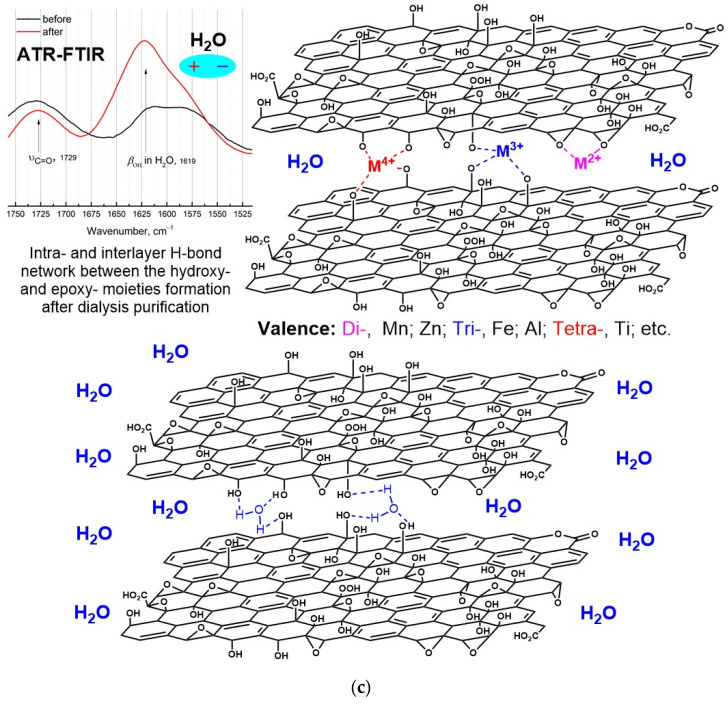
ATR-FTIR spectra of air-dried GO for as-prepared (black line) and after (red line) the dialysis process (3.5 kDa, MWCO). (**a**) Normalized ATR spectra in the range of 4000–100 cm^−1^. (**b**) Band assignments (in cm^−1^). 128 spectra and background scans, 1 cm^−1^ resolution. (**c**) Schematic illustration of the structure and bonds network changes after dialysis purification.

Figure 5 shows attenuated total reflectance Fourier transform infrared spectroscopy (ATR-FTIR) of purified and non-purified GO dispersions. The ATR-FTIR spectrum (Figure 5a) after dialysis demonstrates a series of bands. Band assignments for GO samples are shown in Figure 5b. We distinguish four characteristic ranges: 4000 ÷ 2000, 2000 ÷ 1500, the fingerprint region 1500 ÷ 900, and below 900 cm^−1^.

The intense and broad absorption band in the 3600÷2400 includes water and moieties band ν_O–H_ (3417) [62] and ν_C–H_ (–CH_2_– at 2851 and –C–H at 2938 cm^−1^). All GO samples reveal the primary bands at 1619 (H_2_O), and 1729 cm^−1^ (C=O); overlapping bands in the fingerprint region, which mainly relate to variations in the C–O bond; aromatic C=C at 1584; C–OH or organic carbonates at 1384; C–O–C or covalent ester of H_2_SO_4_ at 1225 cm^−1^; and skeletal modes of C–O and C–C at 1064 and 968 cm^−1^. The hardly assigned short wavenumber region shows bands at 828 (C–O stretching) and 695 cm^−1^ (O–C–C deformation), presumably. The actual IR frequencies are affected by the environment of functional groups. A redshift is observed upon purification for ν_C–O_ 1066→1052, Δν = 14 cm^−1^, and ν_C–O–C_ 1228→1221, Δν = 7 cm^−1^. The band of ν_C–O–H_ appears as a fine structure after dialysis at 1384 cm^−1^. Shifting to higher frequencies (blueshift) for bands of ν_C=C_ 1621→1611, Δν = 10 cm^−1^, and lower frequencies (redshift) ν_C=O_ 1730→1728, Δν = 2 cm^−1^, is registered.

### 3.3. Chemiluminescence Assays

For chemiluminescence measurements, we chose the most purified GO dispersions, which are produced by the dialysis with a 14 kDa MWCO membrane.

#### 3.3.1. SOD-like Activity Using Lucigenin/Xanthine/Xanthine Oxidase System

**Figure 6:**
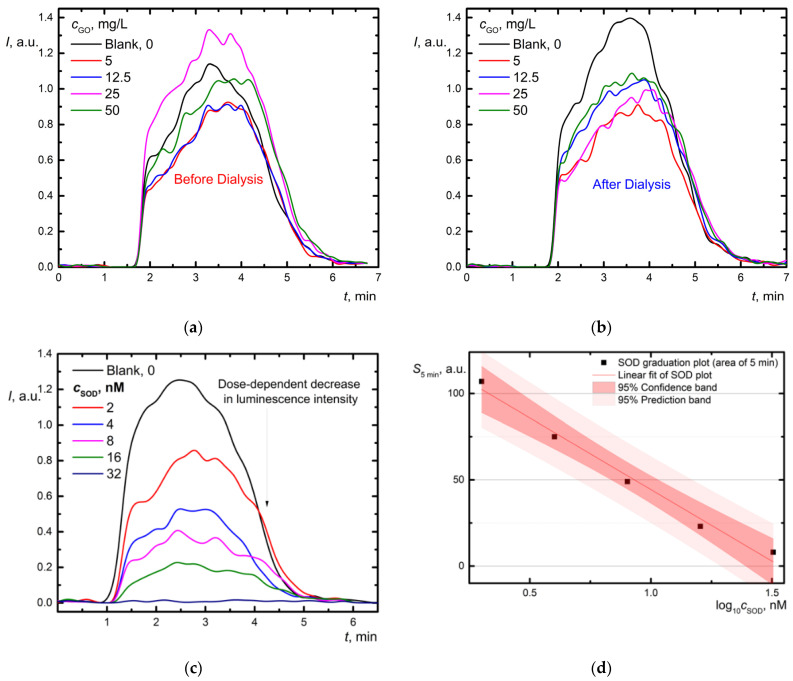
Oxidation of xanthine (20 μM) with xanthine oxidase (4.4 mU/mL) in the presence of lucigenin (20 μM) and GO aqueous dispersions in the range of 5–50 mg/L: (**a**) before dialysis; (**b**) after dialysis; (**c**) source chemiluminograms for SOD; and (**d**) dependence of the light sum (*S*_5min_ is area under curve for 5 min) vs. log_10_*c* (SOD); *S*_5min_ = (−83 ± 6) × log_10_*c* (SOD, nM) + (127 ± 10), *r* = 0.992. Concentrations are shown in the legends. The total volume, 1.00 mL; temperature, 37 °C.

To assess superoxide dismutase-like (SOD-like) activity, the lucigenin/xanthine/xanthine oxidase system was used (Procedure 4). Xanthine oxidase catalyzes the oxidation of xanthine with the formation of superoxide anion. Lucigenin is a highly sensitive and specific probe for superoxide anion. Therefore, measurements were conducted as lucigenin-dependent CL for superoxide anion radical quantification (Figure 6).

Before dialysis, GO samples with low concentrations (5 and 12.5 mg/L) caused luminescence suppression; a higher concentration (25 mg/L) increased chemiluminescence (Figure 6a). After purification, GO in all concentrations had approximately equally reliable suppressed CL signals (Figure 6b). The shape of kinetic curves is similar to chemiluminograms for superoxide dismutase (Figure 6c). The area under the curve reflects the number of free radicals; thus, it is a parameter for constructing a calibration plot (Figure 6d). A dose-dependent decrease in luminescence intensity was observed (Figure 6d). From the calibration, 1 mg/L high-purity GO exhibits a superoxide-intercepting capacity equivalent to 2.00 ± 0.01 nM SOD activity units. Unpurified graphene at 25–50 mg/L did not show antioxidant properties, presumably due to impurities. The absence of a concentration dependence is most likely due to a low sensitivity coefficient or the activity saturation. Thus, GO exhibits antioxidant properties with respect to the superoxide radical. The mechanism of this action should be investigated further.

#### 3.3.2. Catalase/Peroxidase Activity

**Figure 7:**
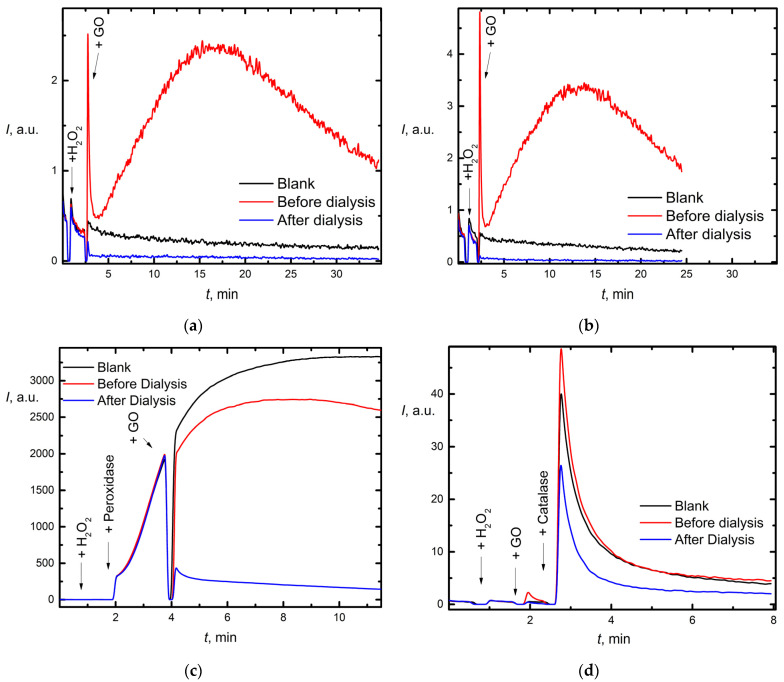
Peroxidase and catalase activities of GO by chemiluminescence with L-012 (40 µL, 1 mM) and H_2_O_2_ (10 µL, 0.1 mM) in PBS pH 7.4. Chemiluminescence kinetics of the model system in presence of (**a**) 1 mg/L aqGO, (**b**) 2.5 mg/L aqGO; (**c**) Peroxidase (20 µL, 0.2 µM) and 1 mg/L aqGO, (**d**) Catalase (10 µL, 7 µM) and 1 mg/L aqGO. The total volume, 1.00 mL; temperature, 37 °C.

In relation to hydrogen peroxide, nanozymes can exhibit either prooxidant (peroxidase) or antioxidant (catalase) activity. The study of peroxidase-like (Px-like) or catalase-like (Cat-like) activity (Procedure 5) was conducted with the L-012/H_2_O_2_ model (Figure 7).

The sensitivity of the L-012 probe is 45-fold higher than that of the luminol model, which is frequently used in CL measurements (Figure S4A, Luminol, and Figure S4B, L-012 probes data, Supplementary Materials). Thus, L-012 was selected as a probe for low concentrations.

Concentration dependence of the CL signal and kinetic behavior of GO in the reaction with H_2_O_2_ is shown in Figure 7a,b. For two concentrations (1 and 2.5 mg/L), similar results were obtained. Before dialysis, GO caused an increase in CL by 5–7 times, thus exhibiting peroxidase-like properties. On the contrary, after dialysis purification, GO suppressed chemiluminescence, thus exhibiting catalase-like properties. It is highly probable that “peroxidase-like” properties of unpurified GO are due to the presence of impurities, particularly metals.

This conclusion can be confirmed by experiments in the presence of native horseradish peroxidase and catalase. Purified graphene oxide sharply (by a factor of five in amplitude) reduces peroxidase chemiluminescence (Figure 7c). Similarly, in the presence of high-purity graphene oxide, the chemiluminescence intensity in the H_2_O_2_ + catalase + L-012 system is almost twofold lower (Figure 7d). Presumably, high-purity graphene oxide quickly neutralizes hydrogen peroxide. A direct effect of GO on enzymes is not excluded, but it is weak, as follows from the analysis of the effect of unpurified graphene.

Thus, the oxidative properties of purified and unpurified GO are essentially different. Unlike commercial graphene oxide, dialysis-purified GO exhibits antioxidant properties to the superoxide anion radical and hydrogen peroxide.

## 4. Discussion

To develop the purification procedure, we used as-prepared GO samples, most common in practice (Procedure 3, Table 1). We aimed to create a setup and developed the procedure to purify aqGO from various contaminants that may be present in various quantities depending on the GO preparation technique [21,63]. Primary contaminants (their quantity depends on preparation techniques) are reagents in Hummers’ method utilizing KMnO_4_, NaNO_3_, and H_2_SO_4_ [64]. Secondary contaminants (their quantity depends on subsequent exfoliating techniques) are substances appearing in ultrasound-assisted exfoliating, which brings forth TiO_2_ as we previously showed for aqueous fullerene dispersions of C_60_, C_70_, and Gd@C_82_ by ultrasound probe operating in a continuous mode [52]. The third type are carbonaceous debris continuously formed during GO synthesis as a byproduct of the main reaction [29]. This requires the development of tools and methodologies for (i) online signals processing to control all the purification stages; (ii) comprehending the endpoint of the dialysis process; (iii) the assessment of purified samples; and (iv) an estimation of biological relevance and applicability of purified GO samples.

### 4.1. Classification of Metal Impurities and Their Removal Mechanism

Evaluation should be done for biomedical applications for in vitro and in vivo purity levels. The most appropriate recommendations with specific minimum requirements for GO dispersions purity are proposed in ISO 23500-3:2019 for water to be used in hemodialysis and related therapies. Results of the inorganic impurity estimation by ICP-AES and IC (Table 1) indicate that we achieved the content of elements below the recommendations in the standard. It is worth noting that Zn (<0.1 ppm) and Al (<0.01 ppm) as well as nitrate (<2 ppm) and sulfate (<100 ppm) contents are significantly reduced during the purification process. In addition, it should be noted that such elements as Fe, Mn, and Ti are not standardized in their contents, although we achieved their reduction to a sub-ppb level.

Ion chromatography shows that sulfate and nitrate have been released in the first two days of the process, which agrees well with the existing data [34], but in case of purifying of carbon dots it should be approx. 15 days [65] due to predominantly random and chaotic carbonization processing [66]. Sulfate in the GO structure can generate an epoxide bridge and a free SO_3_^2−^ radical [26], which retains sulfate in GO samples. As for nitrate, there is no specific role of this anion in the process of GO formation that allowed it to be released completely during the purification.

Due to a high sorption capacity of GO [67,68] and specific metal binding [69], it is unfeasible to conduct the purification by water only. It is necessary to use special reagents; in this work, EDTA was selected as the most used chelation agent and H_2_O_2_ as an oxidation agent. EDTA can bind metals at six points (at two N and four O atoms) and form an elaborate complex with five-membered rings [70].

The regulation of Fe, Mn, and Ti removing deals with chemical transformation to appropriate ions, which are readily removed from GO sheets. We suggest that interlayer ions chelated with GO surface moieties, mainly carboxyl and hydroxyl functional groups, would provide competitive binding with excess EDTA and then released due to diffusion [71]. It is well-known that GO coordinate Mn^2+^ preventing its hydrolysis [72]. It is impossible to unequivocally answer which form of the element is associated with EDTA; the speciation analysis [73] was beyond the scope of this work. A presumable reaction pathway for ions release bonded with GO surface of free ions is presented below.

1. For Fe: ${\mathrm{Fe}}^{2+}/{\mathrm{Fe}}^{3+}\overset{H_{2}O_{2},\;\mathrm{pH}\;<7\;\left(best\;value\;2-3\right)\;\left(\mathrm{for}\;\mathrm{all}\;\mathrm{GO}\;\mathrm{samples}\right)}{\to }\;{\mathrm{Fe}}^{3+}$; then ${\mathrm{Fe}}^{3+}+\mathrm{EDTA}\;\to \mathrm{Fe}\left(\mathrm{III}\right)\cdot \mathrm{EDTA}$; log*K_f_* = 25.1; however, this does not rule out a Fenton’s catalytic pathway [74].
2. For Ti [75]: ${\mathrm{Ti}}^{2+}/{\mathrm{TiO}}^{+}/{\mathrm{TiO}}_{2}\overset{H_{2}O_{2},\;\mathrm{pH}\;3-6}{\to }\;\mathrm{TiO}\left(\mathrm{OH}\right),\mathrm{colourless}$; then ${\mathrm{Ti}}^{3+}+\mathrm{EDTA}\;\to \mathrm{Ti}\left(\mathrm{III}\right)\cdot \mathrm{EDTA}$; log*K_f_* = 21.3.
3. For Mn: ${\mathrm{Mn}}^{2+}/{\mathrm{MnO}}_{2}{\;\mathrm{or}\;\mathrm{Mn}}_{3}O_{4}/{\mathrm{MnO}}_{4}^{-}\left({\mathrm{MnO}}_{3}^{+}\right)\overset{H_{2}O_{2},\;\mathrm{pH}\;<7\;}{\to }{\mathrm{Mn}}^{3+}$.

All stable high-oxidation states (+7, +6, and +4) of Mn reduce to ${\mathrm{Mn}}^{3+}$ species due to comproportionating ${\mathrm{Mn}}^{7+}$ with other Mn oxidation states. ${\mathrm{Mn}}^{3+}$ can be stabilized by EDTA despite ${\mathrm{Mn}}^{2+}$ as a [Mn(H_2_O)_6_]^2+^ is the most stable oxidation state according to a Frost diagram. ${\mathrm{Mn}}^{2+}$ can form a stable EDTA complex only in alkaline media (pH > 8); then ${\mathrm{Mn}}^{3+}+\mathrm{EDTA}\;\to \mathrm{Mn}\left(\mathrm{III}\right)\cdot \mathrm{EDTA}$; log*K_f_* = 25.2. In most cases, manganese is removed with hydrochloric acid solutions, but this affects the stability of GO solutions and requires additional purification for biological applications [76,77]. In some cases, incomplete removal of titanium may have a positive effect, because TiO_2_–GO nanohybrids acquire UV adsorption and radical quenching effects [78].

As for a GO binding site, the presence of carboxyl groups response to metal sorption activity [68,79] and vic-diol transformed to enols can bind divalent metal ions [72]. Presumably, the substantial factor for the high efficiency of EDTA for GO purification is its excess and much more sorption and binding capacities in comparison with GO. Compared with manganese, the EDTA quantity was at a 400-fold excess. As well, for highly oxidized samples, 1 g GO contains 5 to 8 mmol of acidic groups [72], which correlates to the found sub-mmol/g quantity (1.4 ± 0.2 mmol/g), see the Supplementary Materials, Section S1.1 (Figure S5); it is much lower than the EDTA concentration (up to 500 times). Therefore, the thermodynamic equilibrium shifts towards a more stable EDTA complex.

In terms of pore size, 3.5 and 14 kDa membranes correspond to diameters of 2 and 3 nm for proteins, respectively [80]. Regarding graphene oxide flat structure and metal removal efficiency, a 14 kDa membrane provided much better results than a 3.5 kDa membrane (bag). The oxidative debris and metal throughput for a 14 kDa membrane are higher, with the main 1 μm fraction remaining in the solution. Although some approaches have been developed to determine the pore size distribution of nanofiltration membranes [81] for graphene oxide, such techniques have not yet been validated.

### 4.2. Purification Setup Requirements

We propose the following requirements for the setup to carry off the semi-automated purification process. A setup should include the four basic units (Figure 1).

#### 4.2.1. Membrane (Bag)

Currently, dialysis bags [25] or centrifugation–filtration [17] are used to purify GO. A pre-treated membrane (Procedure 2) was used for reducing contaminant contents to prevent their introduction to dialyzed GO samples. According to the manufacturer data, membranes may contain up to 15 ppm of Pb, Zn, and Fe, less than 0.1 ppm of Cd, Cr, Cu, Ni, glycerol to prevent brittleness, and sulfides. EDTA is most appropriate due to the best stability constants for transition metals [82], and H_2_O_2_ is the best reagent for slight oxidation of sulfides, which can be easily removed as free sulfate. Experiments without a pre-treated protocol showed increasing zinc content up to 400% in GO after the dialysis.

In pore-size aqGO separation, the 2 kDa fraction is ~30 wt.% and 2 ÷ 50 kDa < 70 wt.% [83]. Thus, membranes with a molecular weight cut-off (MWCO) 3.5 and 14 kDa were selected to release small inorganic ions, carbonaceous debris, and unoxidized graphite.

#### 4.2.2. Peristaltic Pump for Reproducible Reagent Dosing

Most dialytic purification treatments and approaches use changing water (or reagent solutions) manually and it spreads out the process up to 30 days [25,84]. If the process is unchecked, the endpoint is undetermined. Therefore, we used an automatic reagent supplier using a multichannel peristaltic pump programmable via a Wi-Fi protocol. The pump is needed to deliver the reagents evenly and to regulate the amounts within certain limits. Reducing both quantities and fluxes of reagents are in demand for long-term sustainability of the dialysis process [85]. On average, it takes 25 ÷ 30 L of liquid (pure water and reagents) to purify (approx. 100 mL, up to 0.2 g scale) a GO dispersion in the semi-automation mode for 7 days. The limiting factor in purification and reducing the reagent amounts and their efficiency is the surface area of the dialysis bag [86], which cannot be infinite. The evaluation of the fluid fluxes shows that the process automation ($f_{regents}^{auto}$, L per h) reduces the amounts of reagents for purification by up to 25% compared to manual dialysate replacement ($f_{regents}^{manual}$, L per h), which was not reported previously for GO. Using Float-A-Lyzer™ [87] or Slide-A-Lyzer™ Dialysis Cassettes, e.g., the total area can be increased, but it has not been applied for GO purification yet. Although it is worth noting recent works on the closest topic of carbon dots, where similar membranes are successfully used [65]. As well, a crossflow membrane akin Vivaflow^®^ 50 Laboratory Cross Flow Cassette can increase the purification efficiency but still has not been widely used for GO purification due to the complexity and high operating expense of this process.

Another advantage of gradient-supplying reagents is prevented activation of GO coagulation–flocculation–sedimentation cascades due to the increased ionic strength. Coagulation processes were observed during reagent purification of as-prepared GO samples when reagents were added at once (like HCl, EDTA, etc.) due to disruption of electrostatic interactions and steric repulsion [88].

#### 4.2.3. Real-Time Online Signal Recording

Rapid separation of low molecular weight impurities from GO dispersions requires continuous signal processing of the dialysate. Continuous signal processing would simplify the purification and make it more cost-effective by saving human resources, but not environmentally friendly due to electricity and other resource consumption. Automation of GO purification is essential protocol in high demand on an industrial scale. Attempts have been reported for isolation, purification, and characterization of GO [34] and humic substances [89]; however, not enough methods and devices were used to control the whole process. Attention should be paid to comprehensive automation processes and data collection [90] to reduce: (i) labor-intensive operations, (ii) the duration of the entire process, even while maintaining the number of reagents consumed, (iii) accuracy, and (iv) reproducibility. We suggest a continuous signal registration approach. Previously, merely conductometry monitoring [91] or simultaneous conductivity and pH [27] were used for total online control of dialysis purification in scaling processes [34]. EC is the best option for signal registration because the primary contaminants of GO aqueous dispersions are charged particles. The cheapest option is immersion probes, but their significant disadvantage is the impossibility of continuous operation. The registration of electric conductivity during purification is the most affordable and most accurate option providing the degree of leaching of contaminants (cationic oxidants) and reagents used in dialysis, such as EDTA. However, we propose to use extra ORP and absorbance registration to assess dialysis purification of GO, which makes the process much more efficient. The proposed signal registration provides insight into not only the charged particles (inorganic ions or surface moieties), but also the absorbing particles released during purification, either graphite debris or any other. ORP online registration is a relatively cheap alternative to conductivity measurements. The use of UV/vis probes expands the interpretation of the dialysis process, and this approach has not been used previously for GO purification.

### 4.3. Pros and Contras of Dialysis-Monitoring Methods

UV/vis probes and ORP have not previously been shown to be capable of monitoring dialysis purification of aqueous dispersions. To sum up, these two probes can be considered universal with high accuracy and rapid responses. The practical implementation of these probes provides the maximum economic effect and suitability. To prove that the purification process is over, the analysis of the dialysate for 7-day and 14-day processes shows that all significant contaminants are washed out within the first 48 h of the purification process. The steady-state values of EC, ORP, or absorbance should be achieved within 6 to 12 h; usually, they will be reached on day 3 or day 4 of the process. After that, it is necessary to characterize the prepared sample with physicochemical methods.

#### 4.3.1. ORP Probe

Advantages of ORP monitoring are (i) a wide working temperature range for conducting dialysis between −10 and 100 °C due to the working temperature of Ag|AgCl reference electrode; (ii) the stability of the signal, RSD is lower than 1%; and (iii) visual control and adjustment of all the purification reagent feed fluxes. Adding uncharged hydrogen peroxide lowers ORP by dilution, and adding a charged EDTA, on the contrary, increases the potential due to fluctuation of ionic strength (μ) of the solution, that changes the potential values [92,93].

The main issue in the recording of ORP is the instability (potential difference) for several reasons. The first factor is the noise at low ionic strengths, which correspond to relative conductivity <10 μS/cm [94]. The next is the parasitic leakage current of the power grid [95], which affects the accuracy of measurements [96]. Furthermore, the instability factors are magnetic stirrers and dissolved oxygen and its effect on the platinum electrode [97]. A possible solution to improve the signal quality is a Faraday cage to shield the setup from external electromagnetic fields [98] and the application of uninterruptible power supplies with double conversion (not less than 1500 VA of full power) to improve potential reproducibility [99] and reduce the probability of loss of the registered data [100].

#### 4.3.2. UV/Vis Probe

Continuous measurements require the use of flow cells or spectrophotometric fiber-optic probes. In this work, the UV/vis probe showed high reproducibility of the long-term signal at 254 nm for 10 h, which was ~0.1%. Additionally, the probe accuracy check by a Ferroin solution spectrum and calculation of the apparent molar absorptivity *ε*_510_ = 11,100 ± 200 M^−1^cm^−1^ coincided with existing data [101] (*ε*_510_ = 11,080 M^−1^cm^−1^). Thus, UV/vis probes could be used for washing control from organic reagents, which absorb in 190–400 nm. The dialysate solution was limpid and colorless; the endpoint criteria of absorbance of 0.05 corresponds to <1 ppm of dissolved substances [102].

The disadvantages of UV/vis probe are the instability of operation when air bubbles are formed with poorly degassed solvents, which leads to deviations from Beer’s law and an increased proportion of the scattering component in the absorption spectra.

### 4.4. Characterization of Purified GO Dispersions

Considering the detailed cycle of high-purity GO dispersions preparation from graphite to graphene oxide material and to high-purity graphene oxide aqueous dispersions, each stage requires proper characterization of the resulting sample. These stages include (1) chemical graphite oxidation exfoliation, (2) GO isolation and characterization, (3) extra exfoliation, if any, and further purification and complete characterizations. For the second stage, the methods to verify GO structure using standard protocols [103] are XRD, XPS, SEM, and Raman spectroscopy. Vis-à-vis the third stage, this is much more challenging and requires individual quality standards and criteria. The development trend of such methods and techniques lies in the study of the qualitative and quantitative GO composition of both major and minor, both organic and inorganic impurity components and the analysis of their long-term stability.

For further GO biomedical testing and hemocompatibility, we recommend the chemical information for the three-level characterization of aqGO: (1) elemental composition by ICP-AES, anionic composition by IC and pH-metry; (2) absorption spectra and FTIR for the presence of organic substances or colorants; (3) colloidal stability by DLS. For GO dispersions, we set the water requirements for hemodialysis. Insufficient purity in terms of metal content can set off a clotting cascade [104,105].

The FTIR spectrum of GO dispersions displays the presence of typical GO bands [106]. Several peculiarities deal with absorption bands at 1729 and 1619 cm^−1^, which are distinctive features of any GO sample. There is some ambiguity in the interpretation of the band at 1619 cm^−1^, which could be assigned to C=C [107,108,109]. However, this band corresponds to the absorption of water molecules embedded in graphite layers, proven by isotopic exchange with D_2_O [110,111,112]. As well, dialysis samples showed some FTIR band redshifts up by 30 cm^−1^. More bands in purified samples compared to the unpurified are due to less interaction with contaminants (Figure 5c), which is consistent with the purified proteins [113] and chemical states of edges (in our case purified edges) play a crucial role in electrical transport within graphene-based materials [114].

Higher band intensities at 1619 cm^−1^ are concerned with an increasing quantity of embedded H_2_O dipoles, increased intra- and interlayer H-bonding network between hydroxy- and epoxy- moieties [115] and H-bonds with other water molecules (Figure 5c). These sites play a crucial role as binding sites of removed transition metals. In addition, the GO surface is highly reactive, including epoxide opening and alkoxide shuttling, and their interactions with water as well as proton abstraction events from interfacial waters are possible [116]. The increase in the water band intensities presumably reflects better water sorption capacity owing to shorter diffusion pathways [117] in the purified state of GO sheets. However, more detailed investigations of this sorption capacity are required, that were beyond the scope of this study. As for carbonyl stretching modes at 1729 cm^−1^, its changes are related to the dissociation of the lactone–ether pairs [118].

Raman scattering stands as a standard technique of the quality of GO materials. Raman spectroscopy confirmed the GO structure preservation during the purification process (see the Supplementary Materials, Figure S11). Samples had both G and D vibration bonds of *sp*^2^ carbon. Raman spectra of our materials presented D and G bands centered at about 1330 and 1590 cm^−1^, respectively. Band intensity ratio (*I*_D_/*I*_G_) had the same order of magnitude, but after the dialysis process, increased by 3.5%, which indicated the better alignment of carbon atom structure to a perfect hexagonal honeycomb. In addition, the total area under the curve decreased after the purification process proves changes in samples. The crystallinity structure band D shifted from 1330 to 1339 cm^−1^ (Δ = 9 cm^−1^).

Zeta-potential value is deteriorated after the dialysis process by 20%. For dialysis using both 3 and 14 kDa membranes, zeta-potential shows the same magnitude. The experimental values between −40 to −31 mV correspond to moderate stability (no agglomerates) [119], which correlates to [120]. The lateral size of GO samples stayed the same, slightly increasing after purification. The PDI index has decreased, which tends to be characteristic of monodisperse systems, and the size and shape of the distribution are unchanged.

As a whole, we have achieved significant improvements in the purification process that would provide a broad range of GO applications in the extended phases of purified GO by genotoxicity, cytotoxicity, and biocompatibility tests. The proposed approach to GO characterization by three levels extends the guidelines described elsewhere [121].

### 4.5. Radical Scavenging and Nanozyme Activity of Purified GO Dispersions

#### 4.5.1. Reactivity with Respect to the Superoxide Anion Radical

The main result of experiments in the superoxide anion radical generation model is that highly purified GO exhibits antioxidant properties in all studied concentrations. The suppression of the CL signal (Figure 6b) cannot be explained by enzyme inactivation. GO did not inhibit xanthine oxidase activity that confirms both electrochemically measured uric acid and O_2_ content during the reaction [122]. The formation of uric acid, i.e., xanthine oxidase activity, was not inhibited by any GO samples (the data are not shown).

Thus, after purification, GO possessed significant radical-scavenging activity, which previously was observed for rGO only [123]. Though few-layer-graphene (FLG) or graphene oxide was shown to induce significant oxidative damage at the mitochondrial level consequent to NADH dehydrogenase- and xanthine oxidase-dependent ROS with concentrations from 0.4 to 100 ppm [124], it was commercially available samples without any purification treatment that may be comprised of any contaminants. Thus, when studying the oxidative properties of graphene oxide, it is particularly important to work with highly purified samples to obtain the intrinsic effect of graphene, and not highly free radical-reactive impurities. The presumable action of GO can be related to H-atom donation from –OH, electron transfer [125] from graphene [126], and basal adduct formation [78] as anti-oxidant mechanisms. The graphene-like domain shows highly efficient catalytic conversion of O_2_^•−^ into O_2_ and H_2_O_2_ [127], which becomes more available after the purification. The activity decreases in the row FLG > rGO > GO, mainly caused by the fact that the main active sites are associated with the pristine graphene network [78] rather than with the oxygen-containing functional groups. As we have previously shown for non-functionalized fullerenes [52], the electron affinity in the row C_60_ < C_70_ < Gd@C_82_ plays a crucial role in attaching radicals. However, the quenching effect of chemiluminescence during GO examination should not be underestimated [128].

As the reaction mechanism is complex and multistage, we have limited ourselves to qualitatively comparing the reactivity of purified and unpurified GOs towards superoxide anion radical. Mechanistic studies and quantitative examination of nanozyme SOD-like activity may be the aim of further studies using other methods like EPR, etc.

#### 4.5.2. Reactivity with Respect to Hydrogen Peroxide

Many studies concern nanomaterials with enzyme-like characteristics making of artificial enzymes. Origins of the peroxidase mimicking activities of graphene oxide from first principles were elucidated [129]. GO can activate H_2_O_2_–generating hydroxyl radicals with higher oxidizabilities. Here, unpurified GO samples exhibit much more peroxidase mimicking activities (Figure 7a,b), while purified GO exhibits catalase-like properties. On the one hand, this can be explained by the presence of variable-valence metals (manganese) in solution. On the other hand, this can be justified by a change in the graphene-oxide structure itself. Thus, the authors [130] claimed that carboxyl-modified graphene oxide (GO-COOH) possesses intrinsic peroxidase-like activity that can catalyze the reaction of peroxidase substrate 3,3,5,5-tetramethylbenzidine in the presence of H_2_O_2_, and the observed peroxidase-like activity is not related to the trace metal catalyst in the sample but is caused by its own intrinsic property instead. Efficient analytical systems based on peroxidases and copper ions with GO have been proposed [131,132]. The paper [133] thoroughly considers the possible mechanism of the catalytic action of graphene oxide with respect to hydrogen peroxide. It has been argued that carbonyl C=O is the active peroxidase catalytic center [134], while −COOH is much less effective [129]. The ether is very stable and not a catalytic center; the epoxy, hydroxyl, and endoperoxide are also not catalytic centers. We showed for purified samples that the amount of these acidic centers is much lower than in non-purified samples by titration (see the Supplementary Materials, Section S1.1, Table S2). According to DLS and zeta-potential data, GO lateral size became slightly higher, up to 15% (Table 2) due to slight aggregation activity, which should cause a decrease in activity. On the other hand, compared to large GO nanosheets, GO quantum dots efficiently reduce reactive oxygen species and H_2_O_2_ in vitro and in vivo [135]. Enlarged repulsive interaction by decreasing ionic strength or increasing pH diminish the coagulation of GO platelets [136]. GO samples which may not have been sufficiently purified, which could lead to the formation of small flocculates or aggregates of GO sheets. [137].

Presumably, deep crowding and hydration effects and changes in enzyme conformations [138] are more expressed in purified samples due to much better binding. However, molecular docking and dynamic approaches should be developed further. Some studies show the dual catalytic activity, peroxidase- or catalase-, at different pHs [139]. Here, we studied only physiologically relevant conditions at pH 7.4.

## 5. Conclusions

This study is the first step to developing procedures for estimating and regulating graphene oxide activity. We believe that it is especially essential to extend the study to other types of rGOs or GOs, such as Brodie’s methods, where fewer carboxyl groups and point defects are present [140], for a more rigorous understanding of the catalytic cycle of the process. The developed approach to the purification of GO samples can be adapted to other synthetic carbon nanomaterials (rGO, nanotubes, carbon dots etc.) under bench-scale batches that have significant potential for real-life applications. Highly purified materials would be highly demanded for materials science and in optoelectronics. Challenges ahead also deal with developing a standard purification protocol for carbon-based nanomaterial for biomedical diagnostic purposes. It requires further research on regulating antioxidant or catalytic activity.

Additionally, we believe that an important finding of this study is dialysis-purified graphene oxide itself. Unlike commercial GO, it exhibits antioxidant properties to the superoxide anion radical and hydrogen peroxide, which is of importance for subsequent biocompatibility and toxicity studies. In our opinion, modeling the free-radical kinetics involving purified GO may help draw conclusions about the mechanism of GO action in free-radical models.

It also seems promising to use graphene oxide to create platforms for a remarkable chiral separation (enantioseparation) capacity [141] with an accessible interlayer spacing of 8 Å [142]. Primarily, the dialysis purification would provide specific fractions to create more capacitive sorbents and improve enantiomeric excess.

## Figures and Tables

**Table 1:** Dialysis efficiency of transition metal content purification of GO dispersions estimated by ICP-AES (*n* = 3, *p* = 0.95).

Type of GO Dispersion	Type Reagent for Purification, and Experiment Duration	Concentration, ppb *					Brief Comment
		Mn	Ti	Zn	Fe	Al	
Pristine I series	Not applicable	18,050 ± 900	2450 ± 120	100 ± 10	1050 ± 100	230 ± 10	
After 3.5 kDa, dialysis bag width 18 mm	Only H_2_O, 7 d	25 ± 3	480 ± 35	60 ± 10	220 ± 15	15 ± 3	Almost complete purification from Mn and Al; removes 80% Ti and Fe; does not get rid of Zn, approx. 40%
	EDTA 50 mM, H_2_O_2_ 3% *w*/*v*, 7 d	<2	420 ± 30	65 ± 10	170 ± 20	40 ± 5	Complete purification from Mn; up to 80% reduced Ti, Fe and Al, and 35% Zn content
	EDTA 50 mM, H_2_O_2_ 3% *w*/*v*, 7 d (membrane second usage)	150 ± 15	420 ± 30	45 ± 7	155 ± 20	<2	Repeated dialysis bag usage allows washing out Mn almost completely; Al, completely, decreases Ti and Fe up to 85%; Zn is reduced up to 55%.
Pristine II series	Not applicable	16,080 ± 800	940 ± 70	35 ± 5	750 ± 75	95 ± 10	
After 14 kDa, 25 mm	EDTA 50 mM, H_2_O_2_ 3% *w*/*v*, 7 d	<2	135 ± 15	100 ± 15	125 ± 15	<2	The resulting dispersion contains less Ti due to the replacement of the ultrasonic probe with a new one. The use of a membrane with a higher threshold (MWCO 14 kDa) also completely removes Mn and Al, reduces Ti and Fe contents up to 90%, while the purification increases Zn content up to 300%.
After 14 kDa, 75 mm	EDTA 50 mM, H_2_O_2_ 3% *w*/*v*, 7 d	<2	145 ± 15	20 ± 3	135 ± 15	<2	Complete removal of Mn and Al, reduces Ti and Fe contents up to 85%. and Zn content up to 45%.
	EDTA 50 mM, H_2_O_2_ 3% *w*/*v*, 7 d (membrane second usage)	<2	30 ± 5	30 ± 5	75 ± 10	<2	Complete removal of Mn and Al, reduces Ti and Fe contents up to 97%, and Zn content remains the same.

* The contents of Ag, As, B, Ba, Be, Cd, Co, Cr, Cu, Li, Mo, Ni, Pb, Sb, Se, Sn, Sr, Tl, V, W, Ca, Mg, and Na were below the limits of quantification, <10 ppb.

**Table 2:** Characterization of GO dispersions (*n* = 3, *p* = 0.95).

Type of GO Dispersion	TOC, ppm	Yield, %	pH	$NO_{3}^{-}$, ppm **	$SO_{4}^{2-}$, ppm	Lateral Size, nm	ζ, mV	PDI	κ, µS/cm
Pristine I	2030 ± 100	Not applicable	2.3 ± 0.2	150 ± 25	400 ± 40	1100 ± 120	−36.8 ± 0.5	0.464	2800 ± 140
Pristine, 0.45 µm PTFE filtering	8.0 ± 0.9	0.39	4.4 ± 0.2	n/m	n/m	130 ± 15	−27.5 ± 0.8	0.273	n/m *
3.5 kDa, only H_2_O, 7 d	1710 ± 80	84.2	4.0 ± 0.2	2.0 ± 0.5	78 ± 20	1300 ± 150	−30.4 ± 0.5	0.354	400 ± 40
3.5 kDa, 7 d purification (with reagents)	1650 ± 70	81.3	4.1 ± 0.2	<2.0	45 ± 10	1250 ± 120	−32.6 ± 1.5	0.320	380 ± 40
Reuse of 3.5 kDa, 7 d purification (with reagents)	1320 ± 75	65.0	4.2 ± 0.2	<2.0	40 ± 10	1050 ± 120	−30.6 ± 1.2	0.370	360 ± 45
Boiled membrane preconditioning, 3.5 kDa, 7 d purification (with reagents)	700 ± 50	34.5	4.6 ± 0.2	<2.0	55 ± 10	1270 ± 150	−29.5 ± 1.2	0.370	230 ± 20
Pristine II	1820 ± 120	Not applicable	2.0 ± 0.2	130 ± 15	350 ± 35	1250 ± 130	−37.5 ± 0.5	0.480	2650 ± 120
14 kDa, 7 d purification (with reagents, 25 mm)	877 ± 95	48.2	4.5 ± 0.2	<2.0	15 ± 3	1450 ± 140	−32.5 ± 0.5	0.380	320 ± 45
14 kDa, 7 d purification (with reagents, 75 mm)	1030 ± 130	56.7	4.6 ± 0.2	<2.0	18 ± 4	1375 ± 135	−34.3 ± 0.5	0.390	300 ± 35
Reuse of 14 kDa, 7 d purification (with reagents, 25 mm)	833 ± 80	45.8	4.8 ± 0.2	<2.0	10 ± 2	1550 ± 130	−31.8 ± 0.8	0.370	315 ± 45

* n/m, not measured due to small obtained volumes; **, for F^−^, Cl^−^, Br^−^, NO_2_^−^, PO_4_^3−^, were less <0.2 ppm.

## Data Availability

Not applicable.

## Supplementary Materials

The following Supplementary Materials can be downloaded at: https://www.mdpi.com/article/10.3390/nano12234159/s1, Figure S1: Schematic view of developed in this work dialysis set-up including compartments of sample, stock of reagents, probes, and programmable peristaltic pump.; Figure S2: (A) Absorbance values of dialysate (fraction lower 3.5 kDa) at 254 nm, arrows correspond to changing of reagent; (B) normalized absorbance for GO solution before and after dialysis in bi-logarithmic coordinates.; Figure S3: Normalized ATR-FTIR spectra in a range of 4000 ÷ 100 cm^−1^ (A) and in a range of 1800 ÷ 500 cm^−1^ (B) for pristine membrane (bag) 3.5 kDa (black solid line); pre-treated in EDTA 50 µM (red solid line); pre-treated in water with following dialysis for 7 days (blue solid line); pre-treated in EDTA 50 µM with following dialysis for 6 days (orange solid line).; Figure S4: Calibration plot for (A) Luminol 40 µM; (B) L-012 40 µM probes in H_2_O_2_ (200 µM) different concentrations of peroxidase. Analytical signal was a sum of plot area for 5 min during signal processing. Equations (*n* = 5, *p* = 0.95): Luminol, *S* = (10.4 ± 0.2) × 10^3^ × *c*_Px_, *r* = 0.9917; L-012, *S* = (4.52 ± 0.18) × 10^5^ × *c*_Px_, *r* = 0.9982.; Figure S5: The Derivative End Point Methods for GO samples titration. All dependencies have shown without smoothing. (Black solid line) Diluted, Certified Standard Solutions (CRM) of NaOH; (Red solid line) GO after dialysis, (Blue solid line) GO before dialysis, which have been used to acidity group content estimation.; Figure S6: XPS spectra of bulk graphene oxide sample, which was used in this work.; Figure S7: Dibutyl phthalate (DBP) 1.6 ppm SRM in n-hexane (*n* = 3, *p* = 0.95); Figure S8: Typical chromatogram from EDTA solution. The DBP, DIBP, and DEHP are observed.; Figure S9: Typical chromatogram of H_2_O_2_ solution. The DBP, DIBP, and DEHP are not observed (same as blank solution).; Figure S10: Typical chromatogram of H_2_O solution. The DBP, DIBP, and DEHP are not observed (same as blank solution).; Figure S11: Typical Raman spectra for GO samples (black solid line) after dialysis, (red solid line) before dialysis. Table S1: Conditions for automation of the dialysis process by programmable reagent feeding mode. Example of supply program of the pumps for 24 h operating time (24-h time format), which were then repeated for the required dialysis times. Reagent supply mode: Ch (1) 0.05 M EDTA, (2) 3% *w*/*v* hydrogen peroxide, (3) drain; washing mode: Ch (1,2) water, (3) drain.; Table S2: Quantity of graphene oxide acidity groups estimation; Table S3: C/O ratio estimation by different methods; Table S4: Phthalate estimation by GC-MS. References [143,144,145,146,147,148,149,150,151,152,153,154,155,156,157,158,159] are cited in the Supplementary Materials.

## References

[1] Sun Z., Fang S., Hu Y.H. (2020). 3D Graphene Materials: From Understanding to Design and Synthesis Control. Chem. Rev..

[2] Tene T., Usca G.T., Guevara M., Molina R., Veltri F., Arias M., Caputi L.S., Gomez C.V. (2020). Toward Large-Scale Production of Oxidized Graphene. Nanomaterials.

[3] Jiříčková A., Jankovský O., Sofer Z., Sedmidubský D. (2022). Synthesis and Applications of Graphene Oxide. Materials.

[4] Razaq A., Bibi F., Zheng X., Papadakis R., Jafri S.H.M., Li H. (2022). Review on Graphene-, Graphene Oxide-, Reduced Graphene Oxide-Based Flexible Composites: From Fabrication to Applications. Materials.

[5] Thebo K.H., Qian X., Zhang Q., Chen L., Cheng H.-M., Ren W. (2018). Highly stable graphene-oxide-based membranes with superior permeability. Nat. Commun..

[6] Chen Y.-A., Ou S.-M., Lin C.-C. (2022). Influence of Dialysis Membranes on Clinical Outcomes: From History to Innovation. Membranes.

[7] Karimi K., Rahsepar M. (2022). Optimization of the Urea Removal in a Wearable Dialysis Device Using Nitrogen-Doped and Phosphorus-Doped Graphene. ACS Omega.

[8] Voicu S.I., Thakur V.K. (2021). Graphene-based composite membranes for nanofiltration: Performances and future perspectives. Emergent Mater..

[9] Keramat A.X.A., Kadkhoda J., Farahzadi R., Fathi E., Davaran S. (2022). The potential of Graphene Oxide and reduced Graphene Oxide in diagnosis and treatment of Cancer. Curr. Med. Chem..

[10] Shafiee A., Iravani S., Varma R.S. (2022). Graphene and graphene oxide with anticancer applications: Challenges and future perspectives. MedComm.

[11] Ozkan-Ariksoysal D. (2022). Current Perspectives in Graphene Oxide-Based Electrochemical Biosensors for Cancer Diagnostics. Biosensors.

[12] Oliveira A.M.L., Machado M., Silva G.A., Bitoque D.B., Ferreira J.T., Pinto L.A., Ferreira Q. (2022). Graphene Oxide Thin Films with Drug Delivery Function. Nanomaterials.

[13] Zhihui K., Min D. (2022). Application of Graphene Oxide-Based Hydrogels in Bone Tissue Engineering. ACS Biomater. Sci. Eng..

[14] Ricci A., Cataldi A., Zara S., Gallorini M. (2022). Graphene-Oxide-Enriched Biomaterials: A Focus on Osteo and Chondroinductive Properties and Immunomodulation. Materials.

[15] Zhang J., Wu S., Ma L., Wu P., Liu J. (2020). Graphene oxide as a photocatalytic nuclease mimicking nanozyme for DNA cleavage. Nano Res..

[16] Sun A., Mu L., Hu X. (2017). Graphene Oxide Quantum Dots as Novel Nanozymes for Alcohol Intoxication. ACS Appl. Mater. Interfaces.

[17] Ceriotti G., Romanchuk A.Y., Slesarev A.S., Kalmykov S.N. (2015). Rapid method for the purification of graphene oxide. RSC Adv..

[18] Muhmood T., Cai Z., Lin S., Xiao J., Hu X., Ahmad F. (2020). Graphene/graphitic carbon nitride decorated with AgBr to boost photoelectrochemical performance with enhanced catalytic ability. Nanotechnology.

[19] Muhmood T., Xia M., Lei W., Wang F., Khan M.A. (2018). Design of Graphene Nanoplatelet/Graphitic Carbon Nitride Heterojunctions by Vacuum Tube with Enhanced Photocatalytic and Electrochemical Response. Eur. J. Inorg. Chem..

[20] Pastrana-Martínez L.M., Morales-Torres S., Kontos A.G., Moustakas N.G., Faria J.L., Doña-Rodríguez J.M., Falaras P., Silva A.M. (2013). TiO_2_, surface modified TiO_2_ and graphene oxide-TiO_2_ photocatalysts for degradation of water pollutants under near-UV/Vis and visible light. Chem. Eng. J..

[21] Brisebois P., Siaj M. (2020). Harvesting graphene oxide—Years 1859 to 2019: A review of its structure, synthesis, properties and exfoliation. J. Mater. Chem. C.

[22] De Silva K.K.H., Huang H.-H., Joshi R.K., Yoshimura M. (2017). Chemical reduction of graphene oxide using green reductants. Carbon.

[23] Sun L., Song H., Chang Y., Hou W., Zhang Y., Li H., Han G. (2021). Effective removal of manganese in graphene oxide via competitive ligands and the properties of reduced graphene oxide hydrogels and films. Diam. Relat. Mater..

[24] Kiciński W., Dyjak S. (2020). Transition metal impurities in carbon-based materials: Pitfalls, artifacts and deleterious effects. Carbon.

[25] Chernova E., Petukhov D., Chumakov A., Kirianova A., Sadilov I., Kapitanova O., Boytsova O., Valeev R., Roth S., Eliseev A.A. (2021). The role of oxidation level in mass-transport properties and dehumidification performance of graphene oxide membranes. Carbon.

[26] de Mendonça J.P.A., Lima A.H., Roldao J.C., Martins J.D.S., Junqueira G.M., Quirino W.G., Sato F. (2018). The role of sulfate in the chemical synthesis of graphene oxide. Mater. Chem. Phys..

[27] Bhunia P., Kumar M., De S. (2019). Fast purification of graphene oxide solution by continuous counter current hollow fibre dialysis: A step towards large scale production. Can. J. Chem. Eng..

[28] López-Díaz D., Merchán M.D., Velázquez M.M., Maestro A. (2020). Understanding the Role of Oxidative Debris on the Structure of Graphene Oxide Films at the Air–Water Interface: A Neutron Reflectivity Study. ACS Appl. Mater. Interfaces.

[29] Li X., Yang X., Jia L., Ma X., Zhu L. (2012). Carbonaceous debris that resided in graphene oxide/reduced graphene oxide profoundly affect their electrochemical behaviors. Electrochem. Commun..

[30] Rourke J.P., Pandey P.A., Moore J.J., Bates M., Kinloch I.A., Young R.J., Wilson N.R. (2011). The Real Graphene Oxide Revealed: Stripping the Oxidative Debris from the Graphene-like Sheets. Angew. Chem. Int. Ed..

[31] Jia L., Dong L., Zhu L. (2017). Stripping voltammetry at graphene oxide: The negative effect of carbonaceous debris. Appl. Mater. Today.

[32] Martin-Folgar R., Esteban-Arranz A., Negri V., Morales M. (2022). Toxicological effects of three different types of highly pure graphene oxide in the midge Chironomus riparius. Sci. Total Environ..

[33] Mrózek O., Melounková L., Smržová D., Machálková A., Vinklárek J., Němečková Z., Komárková B., Ecorchard P. (2020). Salt-washed graphene oxide and its cytotoxicity. J. Hazard. Mater..

[34] Tölle F.J., Gamp K., Mülhaupt R. (2014). Scale-up and purification of graphite oxide as intermediate for functionalized graphene. Carbon.

[35] Liu Y., Zhang S., Pei X., Shi H., Li D., Xu Z., Li S., Xue Y., Song L. (2021). Free radical scavenging behavior of multidimensional nanomaterials in γ-irradiated epoxy resin and mechanical and thermal performance of γ-irradiated composites. Compos. Part C Open Access.

[36] Mei Q., Liu B., Han G., Liu R., Han M., Zhang Z. (2019). Graphene Oxide: From Tunable Structures to Diverse Luminescence Behaviors. Adv. Sci..

[37] Mazánek V., Luxa J., Matějková S., Kučera J., Sedmidubský D., Pumera M., Sofer Z. (2019). Ultrapure Graphene Is a Poor Electrocatalyst: Definitive Proof of the Key Role of Metallic Impurities in Graphene-Based Electrocatalysis. ACS Nano.

[38] Tang Z., Zhao L., Yang Z., Liu Z., Gu J., Bai B., Liu J., Xu J., Yang H. (2018). Mechanisms of oxidative stress, apoptosis, and autophagy involved in graphene oxide nanomaterial anti-osteosarcoma effect. Int. J. Nanomed..

[39] Zhang J., Cao H.-Y., Wang J.-Q., Wu G.-D., Wang L. (2021). Graphene Oxide and Reduced Graphene Oxide Exhibit Cardiotoxicity Through the Regulation of Lipid Peroxidation, Oxidative Stress, and Mitochondrial Dysfunction. Front. Cell Dev. Biol..

[40] Mittal S., Kumar V., Dhiman N., Chauhan L.K.S., Pasricha R., Pandey A.K. (2016). Physico-chemical properties based differential toxicity of graphene oxide/reduced graphene oxide in human lung cells mediated through oxidative stress. Sci. Rep..

[41] Srikanth K., Sundar L.S., Pereira E., Duarte A. (2018). Graphene oxide induces cytotoxicity and oxidative stress in bluegill sunfish cells. J. Appl. Toxicol..

[42] Pattammattel A., Williams C.L., Pande P., Tsui W.G., Basu A.K., Kumar C.V. (2015). Biological relevance of oxidative debris present in as-prepared graphene oxide. RSC Adv..

[43] Mazánek V., Matějková S., Sedmidubský D., Pumera M., Sofer Z. (2018). One-Step Synthesis of B/N Co-doped Graphene as Highly Efficient Electrocatalyst for the Oxygen Reduction Reaction: Synergistic Effect of Impurities. Chem. Eur. J..

[44] Ambrosi A., Chee S.Y., Khezri B., Webster R.D., Sofer Z., Pumera M. (2012). Metallic Impurities in Graphenes Prepared from Graphite Can Dramatically Influence Their Properties. Angew. Chem. Int. Ed..

[45] Volkov D.S., Proskurnin M.A., Korobov M.V. (2014). Elemental analysis of nanodiamonds by inductively-coupled plasma atomic emission spectroscopy. Carbon.

[46] Mikheev I.V., Pirogova M.O., Usoltseva L.O., Uzhel A.S., Bolotnik T.A., Kareev I.E., Bubnov V.P., Lukonina N.S., Volkov D.S., Goryunkov A.A. (2021). Green and rapid preparation of long-term stable aqueous dispersions of fullerenes and endohedral fullerenes: The pros and cons of an ultrasonic probe. Ultrason. Sonochem..

[47] Shreiner R., Pratt K. (2004). Standard reference materials: Primary standards and standard reference materials for electrolytic conductivity. NIST Spec. Publ..

[48] Baucke F.G.K. (2002). New IUPAC recommendations on the measurement of pH—Background and essentials. Anal. Bioanal. Chem..

[49] Krivoshein P.K., Volkov D.S., Rogova O.B., Proskurnin M.A. (2022). FTIR Photoacoustic and ATR Spectroscopies of Soils with Aggregate Size Fractionation by Dry Sieving. ACS Omega.

[50] Mikheev I.V., Sozarukova M.M., Izmailov D.Y., Kareev I.E., Proskurnina E.V., Proskurnin M.A. (2021). Antioxidant Potential of Aqueous Dispersions of Fullerenes C_60_, C_70_, and Gd@C_82_. Int. J. Mol. Sci..

[51] Danzer K., Currie L.A. (1998). Guidelines for calibration in analytical chemistry. Part I. Fundamentals and single component calibration (IUPAC Recommendations 1998). Pure Appl. Chem..

[52] Mikheev I.V., Sozarukova M.M., Proskurnina E.V., Kareev I.E., Proskurnin M.A. (2020). Non-Functionalized Fullerenes and Endofullerenes in Aqueous Dispersions as Superoxide Scavengers. Molecules.

[53] Tyurnina A.V., Tzanakis I., Morton J., Mi J., Porfyrakis K., Maciejewska B.M., Grobert N., Eskin D.G. (2020). Ultrasonic exfoliation of graphene in water: A key parameter study. Carbon.

[54] Hall L. (1948). The Origin of Ultrasonic Absorption in Water. Phys. Rev..

[55] Chua C.K., Pumera M. (2015). Light and Atmosphere Affect the Quasi-equilibrium States of Graphite Oxide and Graphene Oxide Powders. Small.

[56] Wang C., Huang P., Qiu C., Li J., Hu S., Sun L., Bai Y., Gao F., Li C., Liu N. (2021). Occurrence, migration and health risk of phthalates in tap water, barreled water and bottled water in Tianjin, China. J. Hazard. Mater..

[57] Auld D.S. (1988). [3] Metal-free dialysis tubing. Methods in Enzymology.

[58] Richmond V.L., Denis R.S., Cohen E. (1985). Treatment of dialysis membranes for simultaneous dialysis and concentration. Anal. Biochem..

[59] Buck R.P., Lindner E. (1994). Recommendations for nomenclature of ionselective electrodes (IUPAC Recommendations 1994). Pure Appl. Chem..

[60] Peacock M., Evans C.D., Fenner N., Freeman C., Gough R., Jones T.G., Lebron I. (2014). UV-visible absorbance spectroscopy as a proxy for peatland dissolved organic carbon (DOC) quantity and quality: Considerations on wavelength and absorbance degradation. Environ. Sci. Process. Impacts.

[61] Saxena S., Tyson T.A., Shukla S., Negusse E., Chen H., Bai J. (2011). Investigation of structural and electronic properties of graphene oxide. Appl. Phys. Lett..

[62] Muhmood T., Xia M., Lei W., Wang F., Mahmood A. (2018). Fe-ZrO2 imbedded graphene like carbon nitride for acarbose (ACB) photo-degradation intermediate study. Adv. Powder Technol..

[63] Chen D., Feng H., Li J. (2012). Graphene Oxide: Preparation, Functionalization, and Electrochemical Applications. Chem. Rev..

[64] Marcano D.C., Kosynkin D.V., Berlin J.M., Sinitskii A., Sun Z., Slesarev A., Alemany L.B., Lu W., Tour J.M. (2010). Improved Synthesis of Graphene Oxide. ACS Nano.

[65] González-González R.B., González L.T., Madou M., Leyva-Porras C., Martinez-Chapa S.O., Mendoza A. (2022). Synthesis, Purification, and Characterization of Carbon Dots from Non-Activated and Activated Pyrolytic Carbon Black. Nanomaterials.

[66] Liang W., Ge L., Hou X., Ren X., Yang L., Bunker C.E., Overton C.M., Wang P., Sun Y.-P. (2019). Evaluation of Commercial “Carbon Quantum Dots” Sample on Origins of Red Absorption and Emission Features. C.

[67] Peng W., Li H., Liu Y., Song S. (2017). A review on heavy metal ions adsorption from water by graphene oxide and its composites. J. Mol. Liq..

[68] Klímová K., Pumera M., Luxa J., Jankovský O., Sedmidubský D., Matějková S., Sofer Z. (2016). Graphene Oxide Sorption Capacity toward Elements over the Whole Periodic Table: A Comparative Study. J. Phys. Chem. C.

[69] Amirov R.R., Shayimova J., Nasirova Z., Solodov A., Dimiev A.M. (2018). Analysis of competitive binding of several metal cations by graphene oxide reveals the quantity and spatial distribution of carboxyl groups on its surface. Phys. Chem. Chem. Phys..

[70] Atkins P., Overton T. (2010). Shriver and Atkins’ Inorganic Chemistry.

[71] Kharisov B.I., Kharissova O.V., Dimas A.V., De La Fuente I.G., Méndez Y.P. (2016). Review: Graphene-supported coordination complexes and organometallics: Properties and applications. J. Co-ord. Chem..

[72] Shayimova J., Amirov R.R., Iakunkov A., Talyzin A., Dimiev A.M. (2021). Carboxyl groups do not play the major role in binding metal cations by graphene oxide. Phys. Chem. Chem. Phys..

[73] Quiroz W. (2021). Speciation analysis in chemistry. ChemTexts.

[74] Walling C. (1975). Fenton’s reagent revisited. Accounts Chem. Res..

[75] Mori M., Shibata M., Kyuno E., Ito S. (1956). Reaction of Hydrogen Peroxide with Titanium (IV) at Different pH Values. Bull. Chem. Soc. Jpn..

[76] Sitko R., Turek E., Zawisza B., Malicka E., Talik E., Heimann J., Gagor A., Feist B., Wrzalik R. (2013). Adsorption of divalent metal ions from aqueous solutions using graphene oxide. Dalton Trans..

[77] Yang X., Zhou T., Ren B., Hursthouse A., Zhang Y. (2018). Removal of Mn (II) by Sodium Alginate/Graphene Oxide Composite Double-Network Hydrogel Beads from Aqueous Solutions. Sci. Rep..

[78] Qiu Y., Wang Z., Owens A.C.E., Kulaots I., Chen Y., Kane A.B., Hurt R.H. (2014). Antioxidant chemistry of graphene-based materials and its role in oxidation protection technology. Nanoscale.

[79] Zhao G., Li J., Ren X., Chen C., Wang X. (2011). Few-Layered Graphene Oxide Nanosheets As Superior Sorbents for Heavy Metal Ion Pollution Management. Environ. Sci. Technol..

[80] Erickson H.P. (2009). Size and Shape of Protein Molecules at the Nanometer Level Determined by Sedimentation, Gel Filtration, and Electron Microscopy. Biol. Proced. Online.

[81] Sutariya B., Karan S. (2022). A realistic approach for determining the pore size distribution of nanofiltration membranes. Sep. Purif. Technol..

[82] Anderegg G. (2013). Critical Survey of Stability Constants of EDTA Complexes: Critical Evaluation of Equilibrium Constants in Solution: Stability Constants of Metal Complexes.

[83] Matsumoto I., Sekiya R., Haino T. (2019). A protocol for size separation of nanographenes. RSC Adv..

[84] Li J., Cui R., Chang Y., Guo X., Gu W., Huang H., Chen K., Lin G., Dong J., Xing G. (2016). Adaption of the structure of carbon nanohybrids toward high-relaxivity for a new MRI contrast agent. RSC Adv..

[85] Agar J.W.M., Barraclough K.A. (2020). Water use in dialysis: Environmental considerations. Nat. Rev. Nephrol..

[86] Abe M., Masakane I., Wada A., Nakai S., Nitta K., Nakamoto H. (2021). Dialyzer surface area is a significant predictor of mortality in patients on hemodialysis: A 3-year nationwide cohort study. Sci. Rep..

[87] D’Souza S.S., DeLuca P.P. (2005). Development of a dialysis in vitro release method for biodegradable microspheres. AAPS PharmSciTech.

[88] Zou Y., Wang X., Ai Y., Liu Y., Li J., Ji Y., Wang X. (2016). Coagulation Behavior of Graphene Oxide on Nanocrystallined Mg/Al Layered Double Hydroxides: Batch Experimental and Theoretical Calculation Study. Environ. Sci. Technol..

[89] Van Zomeren A., Van Der Weij-Zuiver E., Comans R.N.J. (2008). Development of an automated system for isolation and purification of humic substances. Anal. Bioanal. Chem..

[90] Love J. (2007). Process Automation Handbook: A Guide to Theory and Practice.

[91] Bhunia P., Kumar M., De S. (2019). Rapid and efficient removal of ionic impurities from graphene oxide through hollow fiber diafiltration. Sep. Purif. Technol..

[92] Ding X., Scieszka D., Watzele S., Xue S., Garlyyev B., Haid R.W., Bandarenka A.S. (2022). A Systematic Study of the Influence of Electrolyte Ions on the Electrode Activity. ChemElectroChem.

[93] Brandariz I., Vilariño T., Alonso P., Herrero R., Fiol S., de Vicente M.E.S. (1998). Effect of ionic strength on the formal potential of the glass electrode in various saline media. Talanta.

[94] Bagshaw E.A., Wadham J.L., Tranter M., Beaton A.D., Hawkings J.R., Lamarche-Gagnon G., Mowlem M.C. (2021). Measuring pH in low ionic strength glacial meltwaters using ion selective field effect transistor (ISFET) technology. Limnol. Oceanogr. Methods.

[95] Hauschild W., Lemke E. (2018). High-Voltage Test and Measuring Techniques.

[96] Texas Instruments (2008). AN-1852 Designing with pH Electrodes.

[97] Warner T.B., Schuldiner S. (1965). Effects of Oxygen Absorbed in the Skin of a Platinum Electrode on the Determination of Carbon Monoxide Adsorption. J. Phys. Chem..

[98] Valenzuela A.C., de la Rosa J.F.V., Salas R.M., Nájera S.S., Medina L. (2021). Design of a faraday cage for biomedical measurements based on site electromagnetic field mapping. AIP Conf. Proc..

[99] Katarzyński J., Olesz M. (2020). Fault Loop Impedance Measurement in Circuits Fed by UPS and Principle of Safety Protection. Sustainability.

[100] Winkler I., Gomes A.T., Winkler I., Gomes A.T. (2017). Chapter 10—Countermeasures. Advanced Persistent Security.

[101] Jeffery P.G., Hutchison D., Hutchison D. (1981). Chemical Methods of Rock Analysis.

[102] Grieve I.C. (1985). Determination of dissolved organic matter in streamwater using visible spectrophotometry. Earth Surf. Process. Landforms.

[103] Zhang Z., Schniepp H.C., Adamson D.H. (2019). Characterization of graphene oxide: Variations in reported approaches. Carbon.

[104] Feng R., Yu Y., Shen C., Jiao Y., Zhou C. (2015). Impact of graphene oxide on the structure and function of important multiple blood components by a dose-dependent pattern. J. Biomed. Mater. Res. Part A.

[105] Palmieri V., Perini G., De Spirito M., Papi M. (2019). Graphene oxide touches blood: In vivo interactions of bio-coronated 2D materials. Nanoscale Horiz..

[106] Ossonon B.D., Bélanger D. (2017). Synthesis and characterization of sulfophenyl-functionalized reduced graphene oxide sheets. RSC Adv..

[107] Emiru T.F., Ayele D.W. (2017). Controlled synthesis, characterization and reduction of graphene oxide: A convenient method for large scale production. Egypt. J. Basic Appl. Sci..

[108] Prodan D., Moldovan M., Furtos G., Saroși C., Filip M., Perhaița I., Carpa R., Popa M., Cuc S., Varvara S. (2021). Synthesis and Characterization of Some Graphene Oxide Powders Used as Additives in Hydraulic Mortars. Appl. Sci..

[109] Guo H.-L., Wang X.-F., Qian Q.-Y., Wang F.-B., Xia X.-H. (2009). A Green Approach to the Synthesis of Graphene Nanosheets. ACS Nano.

[110] Szabó T., Berkesi O., Dékány I. (2005). DRIFT study of deuterium-exchanged graphite oxide. Carbon.

[111] Szabó T., Berkesi O., Forgó P., Josepovits K., Sanakis Y., Petridis D., Dékány I. (2006). Evolution of Surface Functional Groups in a Series of Progressively Oxidized Graphite Oxides. Chem. Mater..

[112] Dimiev A.M., Alemany L.B., Tour J.M. (2013). Graphene Oxide. Origin of Acidity, Its Instability in Water, and a New Dynamic Structural Model. ACS Nano.

[113] Lorenz-Fonfria V.A. (2020). Infrared Difference Spectroscopy of Proteins: From Bands to Bonds. Chem. Rev..

[114] Kempiński M., Florczak P., Jurga S., Śliwińska-Bartkowiak M., Kempiński W. (2017). The impact of adsorption on the localization of spins in graphene oxide and reduced graphene oxide, observed with electron paramagnetic resonance. Appl. Phys. Lett..

[115] Medhekar N.V., Ramasubramaniam A., Ruoff R.S., Shenoy V.B. (2010). Hydrogen Bond Networks in Graphene Oxide Composite Paper: Structure and Mechanical Properties. ACS Nano.

[116] David R., Tuladhar A., Zhang L., Arges C.G., Kumar R. (2020). Effect of Oxidation Level on the Interfacial Water at the Graphene Oxide–Water Interface: From Spectroscopic Signatures to Hydrogen-Bonding Environment. J. Phys. Chem. B.

[117] Lian B., De Luca S., You Y., Alwarappan S., Yoshimura M., Sahajwalla V., Smith S.C., Leslie G., Joshi R.K. (2018). Extraordinary water adsorption characteristics of graphene oxide. Chem. Sci..

[118] Larciprete R., Fabris S., Sun T., Lacovig P., Baraldi A., Lizzit S. (2011). Dual Path Mechanism in the Thermal Reduction of Graphene Oxide. J. Am. Chem. Soc..

[119] Schramm L.L. (2005). Colloid Stability. Emulsions, Foams, and Suspensions.

[120] Baskoro F., Wong C.-B., Kumar S.R., Chang C.-W., Chen C.-H., Chen D.W., Lue S.J. (2018). Graphene oxide-cation interaction: Inter-layer spacing and zeta potential changes in response to various salt solutions. J. Membr. Sci..

[121] Reina G., González-Domínguez J.M., Criado A., Vázquez E., Bianco A., Prato M. (2017). Promises, facts and challenges for graphene in biomedical applications. Chem. Soc. Rev..

[122] Lakshmi D., Whitcombe M., Davis F., Sharma P.S., Prasad B.B. (2011). Electrochemical Detection of Uric Acid in Mixed and Clinical Samples: A Review. Electroanalysis.

[123] Tayade U.S., Borse A.U., Meshram J.S. (2019). Green reduction of graphene oxide and its applications in band gap calculation and antioxidant activity. Green Mater..

[124] Pelin M., Fusco L., Martín C., Sosa S., Frontiñán-Rubio J., González-Domínguez J.M., Durán-Prado M., Vázquez E., Prato M., Tubaro A. (2018). Graphene and graphene oxide induce ROS production in human HaCaT skin keratinocytes: The role of xanthine oxidase and NADH dehydrogenase. Nanoscale.

[125] Mahmudzadeh M., Yari H., Ramezanzadeh B., Mahdavian M. (2019). Urtica dioica extract as a facile green reductant of graphene oxide for UV resistant and corrosion protective polyurethane coating fabrication. J. Ind. Eng. Chem..

[126] Pathipati S.R., Pavlica E., Treossi E., Palermo V., Bratina G. (2020). The role of charge transfer at reduced graphene oxide/organic semiconductor interface on the charge transport properties. Org. Electron..

[127] Jalilov A.S., Nilewski L.G., Berka V., Zhang C., Yakovenko A.A., Wu G., Kent T.A., Tsai A.-L., Tour J.M. (2017). Perylene Diimide as a Precise Graphene-like Superoxide Dismutase Mimetic. ACS Nano.

[128] Creighton M.A., Rangel-Mendez J.R., Huang J., Kane A.B., Hurt R.H. (2013). Graphene-Induced Adsorptive and Optical Artifacts During In Vitro Toxicology Assays. Small.

[129] Wang D., Song X., Li P., Gao X.J., Gao X. (2020). Origins of the peroxidase mimicking activities of graphene oxide from first principles. J. Mater. Chem. B.

[130] Song Y., Qu K., Zhao C., Ren J., Qu X. (2010). Graphene Oxide: Intrinsic Peroxidase Catalytic Activity and Its Application to Glucose Detection. Adv. Mater..

[131] Wang S., Cazelles R., Liao W.-C., Vázquez-González M., Zoabi A., Abu-Reziq R., Willner I. (2017). Mimicking Horseradish Peroxidase and NADH Peroxidase by Heterogeneous Cu^2+^-Modified Graphene Oxide Nanoparticles. Nano Lett..

[132] Li F., Ma W., Liu J., Wu X., Wang Y., He J. (2018). Luminol, horseradish peroxidase, and glucose oxidase ternary functionalized graphene oxide for ultrasensitive glucose sensing. Anal. Bioanal. Chem..

[133] Su C., Acik M., Takai K., Lu J., Hao S.-j., Zheng Y., Wu P., Bao Q., Enoki T., Chabal Y.J. (2012). Probing the catalytic activity of porous graphene oxide and the origin of this behaviour. Nat. Commun..

[134] Nelson D.L., Lehninger A.L., Cox M.M. (2008). Lehninger Principles of Biochemistry.

[135] Ren C., Hu X., Zhou Q. (2018). Graphene Oxide Quantum Dots Reduce Oxidative Stress and Inhibit Neurotoxicity In Vitro and In Vivo through Catalase-Like Activity and Metabolic Regulation. Adv. Sci..

[136] Kim J.E., Han T.H., Lee S.H., Kim J.Y., Ahn C.W., Yun J.M., Kim S.O. (2011). Graphene Oxide Liquid Crystals. Angew. Chem. Int. Ed..

[137] Koltonow A.R., Luo C., Luo J., Huang J. (2017). Graphene Oxide Sheets in Solvents: To Crumple or Not To Crumple?. ACS Omega.

[138] Bai Y., Ming Z., Cao Y., Feng S., Yang H., Chen L., Yang S.-T. (2017). Influence of graphene oxide and reduced graphene oxide on the activity and conformation of lysozyme. Colloids Surf. B Biointerfaces.

[139] Song L., Huang C., Zhang W., Ma M., Chen Z., Gu N., Zhang Y. (2016). Graphene oxide-based Fe2O3 hybrid enzyme mimetic with enhanced peroxidase and catalase-like activities. Colloids Surf. A Physicochem. Eng. Asp..

[140] Talyzin A.V., Mercier G., Klechikov A., Hedenström M., Johnels D., Wei D., Cotton D., Opitz A., Moons E. (2017). Brodie vs. Hummers graphite oxides for preparation of multi-layered materials. Carbon.

[141] Li X., Tong X., Chen Q., Liu H. (2021). Size effect of graphene oxide sheets on enantioseparation performances in membrane separation. Colloids Surf. A Physicochem. Eng. Asp..

[142] Liu J., Yuan W., Li C., Cheng M., Su Y., Xu L., Chu T., Hou S. (2021). l-Cysteine-Modified Graphene Oxide-Based Membrane for Chiral Selective Separation. ACS Appl. Mater. Interfaces.

[143] Guo S., Zhang G., Guo Y., Yu J.C. (2013). Graphene oxide–Fe2O3 hybrid material as highly efficient heterogeneous catalyst for degradation of organic contaminants. Carbon.

[144] Matsumura Y., Hagiwara S., Takahashi H. (1976). Automatic potentiometric titration of surface acidity of carbon black. Carbon.

[145] Sun C., Berg J.C. (2003). A review of the different techniques for solid surface acid-base characterization. Adv. Colloid Interface Sci..

[146] Langley L.A., Villanueva E.D., Fairbrother D.H. (2006). Quantification of Surface Oxides on Carbonaceous Materials. Chem. Mater..

[147] Szabó T., Tombácz E., Illés E., Dekany I. (2004). Enhanced acidity and pH-dependent surface charge characterization of successively oxidized graphite oxides. Carbon.

[148] Long C.M., Nascarella M.A., Valberg P.A. (2013). Carbon black vs. black carbon and other airborne materials containing elemental carbon: Physical and chemical distinctions. Environ. Pollut..

[149] Zhang Z., Flaherty D.W. (2020). Modified potentiometric titration method to distinguish and quantify oxygenated functional groups on carbon materials by pKa and chemical reactivity. Carbon.

[150] Linstorm P. (1998). Nist chemistry webbook, nist standard reference database number 69. J. Phys. Chem. Ref. Data Monogr..

[151] Park S., An J., Jung I., Piner R.D., An S.J., Li X., Velamakanni A., Ruoff R.S. (2009). Colloidal Suspensions of Highly Reduced Graphene Oxide in a Wide Variety of Organic Solvents. Nano Lett..

[152] Brodie B.C. (1859). XIII. On the atomic weight of graphite. Philos. Trans. R. Soc. Lond..

[153] Staudenmaier L. (1898). Verfahren zur Darstellung der Graphitsäure. Ber. Dtsch. Chem. Ges..

[154] Hofmann U., König E. (1937). Untersuchungen über Graphitoxyd. Z. Für Anorg. Und Allg. Chem..

[155] Hummers W.S., Offeman R.E. (1958). Preparation of Graphitic Oxide. J. Am. Chem. Soc..

[156] Farivar F., Lay Yap P., Karunagaran R.U., Losic D. (2021). Thermogravimetric Analysis (TGA) of Graphene Materials: Effect of Particle Size of Graphene, Graphene Oxide and Graphite on Thermal Parameters. C.

[157] Abdolhosseinzadeh S., Asgharzadeh H., Seop Kim H. (2015). Fast and fully-scalable synthesis of reduced graphene oxide. Sci. Rep..

[158] Shao G., Lu Y., Wu F., Yang C., Zeng F., Wu Q. (2012). Graphene oxide: The mechanisms of oxidation and exfoliation. J. Mater. Sci..

[159] Sengupta I., Sharat Kumar S.S.S., Pal S.K., Chakraborty S. (2020). Characterization of structural transformation of graphene oxide to reduced graphene oxide during thermal annealing. J. Mater. Res..

