# Supplementary material for: High-Throughput Preparation of Uncontaminated Graphene-Oxide Aqueous Dispersions with Antioxidant Properties by Semi-Automated Diffusion Dialysis
Source: Nanomaterials (Basel). 2022 Nov 24;12(23):4159. doi: 10.3390/nano12234159 (PMC9739863; doi:10.3390/nano12234159)
Supplement: Supplementary file 1 [file nanomaterials-12-04159-s001.zip › nanomaterials-1995936-supplementary.pdf]

Supplementary Materials

High-Throughput Preparation of Uncontaminated Graphene-Oxide Aqueous Dispersions with Antioxidant Properties by Semi-Automated Diffusion Dialysis

Ivan V. Mikheev [1,*], Sofiya M. Byvsheva [1], Madina M. Sozarukova [1,2], Sergey Yu. Kottsov [2], Elena V. Proskurnina [3] and Mikhail A. Proskurnin [1,*]

[1] Department of Chemistry, Lomonosov Moscow State University, Moscow 119991, Russia
[2] Kurnakov Institute of General and Inorganic Chemistry, Russian Academy of Sciences, Moscow 117901, Russia
[3] Research Centre for Medical Genetics, Moscow 115522, Russia
* Correspondence: mikheev.ivan@gmail.com (I.V.M.); proskurnin@gmail.com (M.A.P.); Tel.: +7-495-939-15-68 (I.V.M.)

Table of Contents

Figures **2**
Dialysis set-up 2
Absorbance spectra 3
FTIR of membrane (bag) degradation 4
Enzymes activity evaluations 5

Tables **6**
Programmatic peristaltic pump regimes 6

1. Extra experiments information **7**
1.1 Quantity of acidic groups estimation 7
1.2 Finding graphene oxide surface moieties by X-ray Photoelectron Spectroscopy (XPS) 11
1.3 XPS accuracy estimation by gravimetry 14
1.4 Pre-soaking regent's tanks. Estimation of plasticizer quantity. 16
1.5 Raman spectra for GO sample characterisation 20

Supplementary References List **22**

Figures

Dialysis set-up

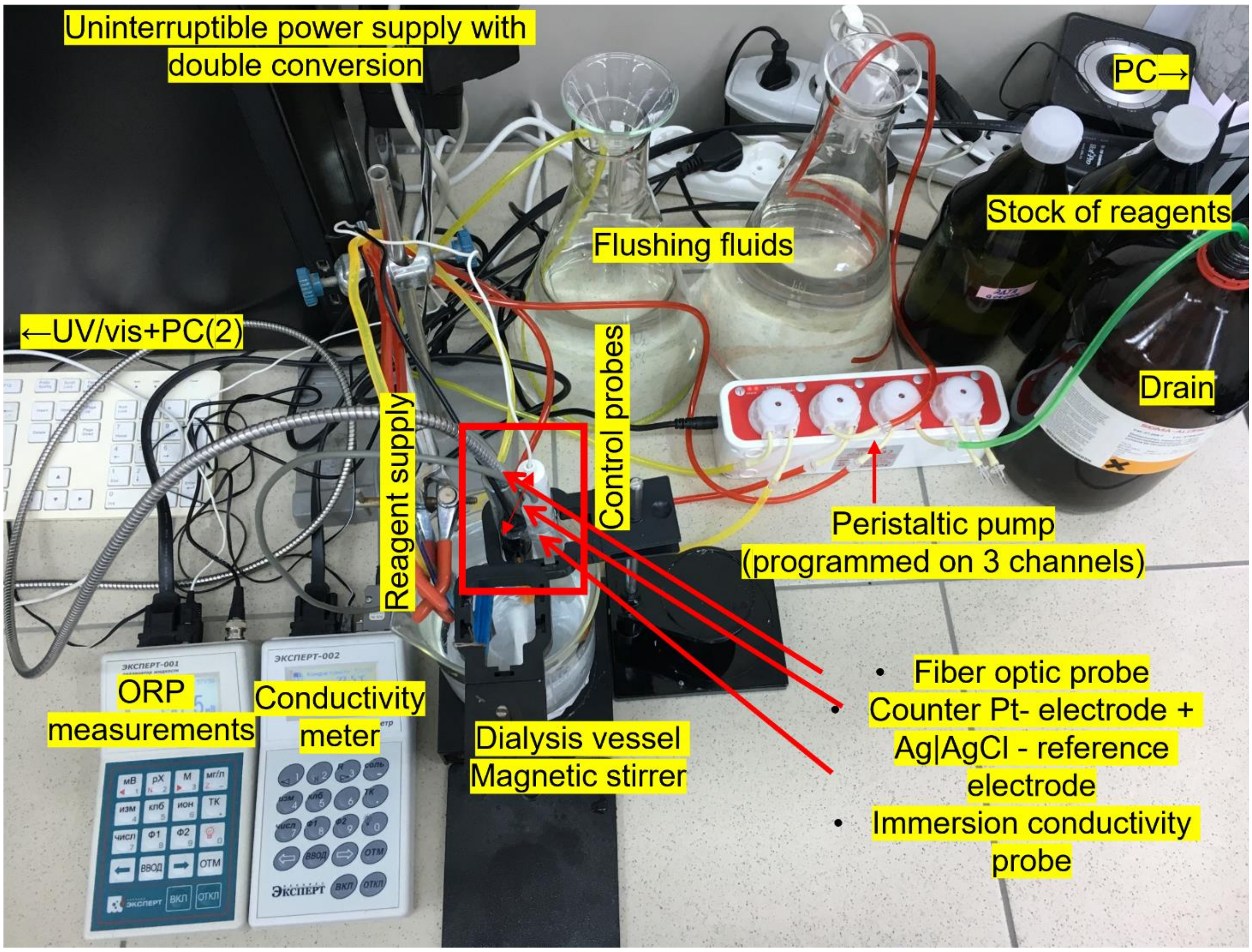

Figure S1: Schematic view of the dialysis setup developed in this work, including compartments of sample, stock of reagents, probes, and a programmable peristaltic pump.

Absorbance spectra

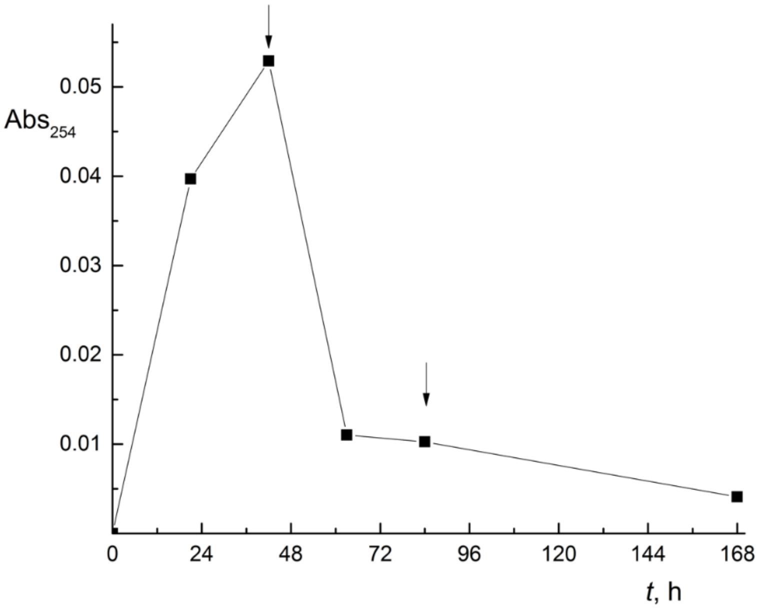

(A)

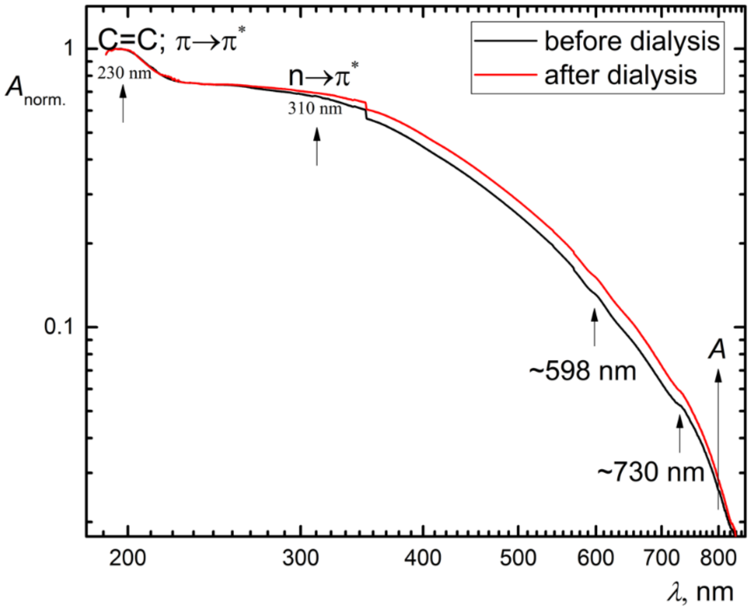

(B)

Figure S2: (**A**) Absorbance values of dialysate (fraction lower 3.5 kDa) at 254 nm, arrows correspond to changing of reagents; (**B**) normalized absorbance for GO solution before and after dialysis in bi-logarithmic coordinates.

FTIR of membrane (bag) degradation

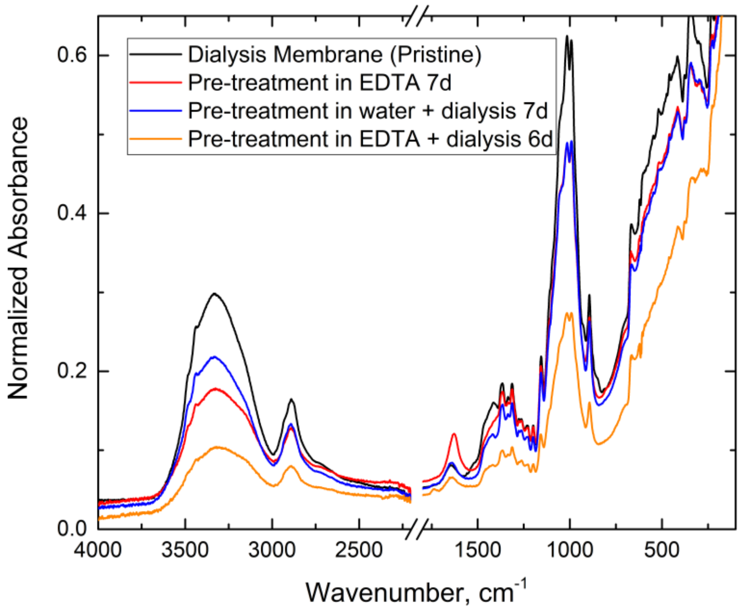

(A)

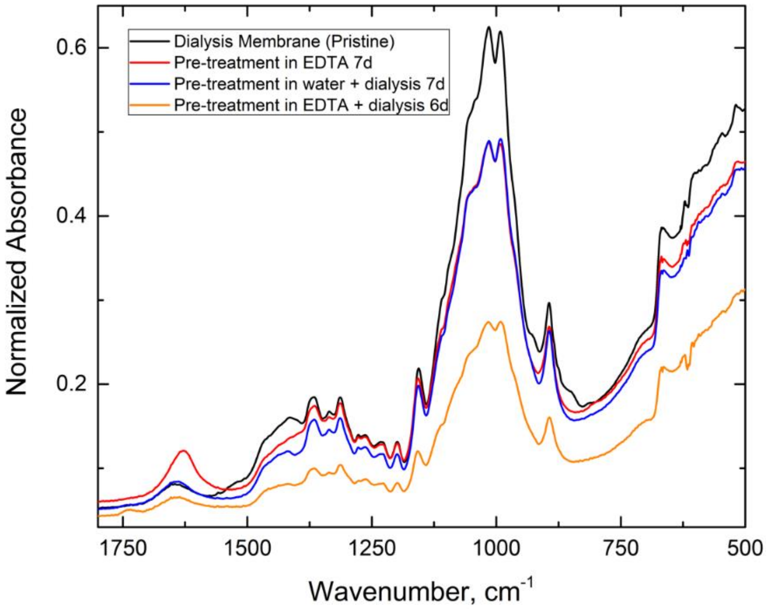

(B)

Figure S3: Normalized ATR-FTIR spectra in a range of 4000÷100 cm^{-1} (A) and in a range of 1800÷500 cm^{-1} (B) for pristine membrane (bag) 3.5 kDa (black solid line); pre-treated in EDTA 50 μM (red solid line); pre-treated in water with following dialysis for 7 days (blue solid line); pre-treated in EDTA 50 μM with following dialysis for 6 days (orange solid line).

Enzymes activity evaluations

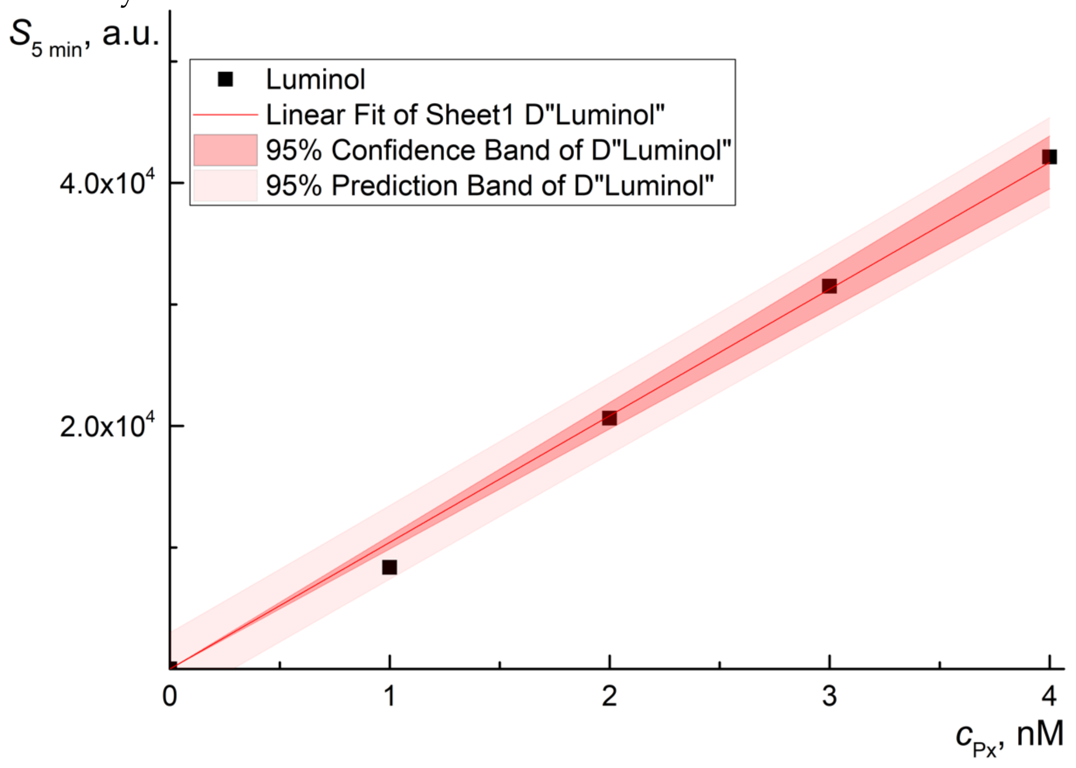

(A)

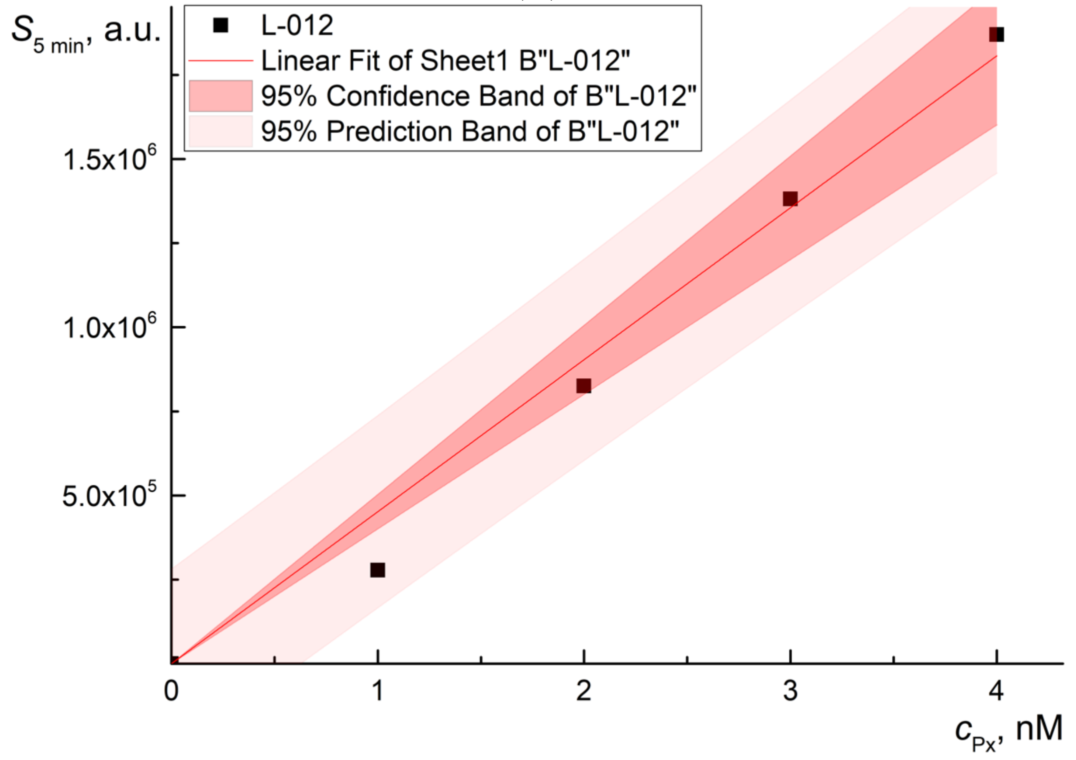

(B)

Figure S4: Calibration plot for (**A**) Luminol 40 μM; (**B**) L-012 40 μM probes in H_2O_2 (200 μM), different concentrations of peroxidase. Analytical signal was a sum of plot area for 5 min during signal processing.

Equations (n=5, P=0.95):

- Luminol, $S=(10.4 \pm 0.2)\times10^3\times c_{Px}$, r=0.9917;
- L-012, $S=(4.52 \pm 0.18)\times10^5\times c_{Px}$, r=0.9982.

Tables

Programmatic peristaltic pump regimes

Table S1: Conditions for automation of the dialysis process by programmable reagent feeding mode. Example of supply program of the pumps for 24 h operating time (24-hour time format), which were then repeated for the required dialysis times. Reagent supply mode: Ch (1) 0.05 M EDTA, (2) 3% w/v hydrogen peroxide, (3) drain; washing mode: Ch (1,2) water, (3) drain.

Cycle number	Time Period	Used pump channel number
1	14:00	1
	14:30	3
	15:00	2
	15:30	3
2	16:00	1
	16:30	3
	17:00	2
	17:30	3
3	18:00	1
	18:30	3
	19:00	2
	19:30	3
4	20:00	1
	20:30	3
	21:00	2
	21:30	3
5	22:00	1
	22:30	3
	23:00	2
	23:30	3
6	0:00	1
	0:30	3
	1:00	2
	1:30	3
7	2:00	1
	2:30	3
	3:00	2
	3:30	3
8	4:00	1
	4:30	3
	5:00	2
	5:30	3
9	6:00	1
	6:30	3
	7:00	2
	7:30	3
10	8:00	1
	8:30	3
	9:00	2
	9:30	3
11	10:00	1
	10:30	3
	11:00	2
	11:30	3
12	12:00	1
	12:30	3
	13:00	2
	13:30	3

1. Extra experiments information

1.1 Quantity of acidic groups estimation

Graphene oxide (GO) is a quasi-two-dimensional material with carbon atoms in the sp^2-(graphene sites) and sp^3-hybridization state (oxidized regions of graphene). At that, hydroxyl and epoxide groups dominate in the graphene oxide surface plane, while carboxyl, phenolic, lactone, and quinone groups are localized at the edges of the layers [1]. Due to the presence of surface groups, OH has hydrophilic properties and dissolves (disperses) well in aqueous solution. Acid groups on carbon-containing materials can be divided into two classes: stronger acids with pK_a 7.0 (including carboxylic acids) and weaker acids with pK_a 7.0 (including hydroxyl groups). It has been noted that the dissociation constant values of the carboxylic groups on the OH surface are not identical; indeed, they have been found to vary depending on the neighboring groups or the position of the groups [2].

There are many methods for measuring the surface acidity of materials, namely titration in both aqueous and non-aqueous media, calorimetry [3] and X-ray photoelectron spectroscopy [4]. Acid–alkali titration is a simple method that is used both to quantify the surface of acidic groups [5] and to study the proton- or metal-binding properties of carbon-containing materials.

Boehm titration is a quantitative analysis of functional groups on the surface of carbon materials based on the assumption that different types of groups can be neutralized by different types of bases with different basicity $NaHCO_3$ (pK_a = 6.4), Na_2CO_3 (pK_a = 10.3) and NaOH (pK_a = 15.7). Boehm titration was originally developed for carbonaceous materials such as conductive carbon black (CCB) [6], activated carbon, porous carbon, and

graphite. Modern carbon-based materials such as graphene, graphene oxide, or carbon nanotubes can also be analyzed in the same way.

The essence of the method is that a sample of the substance is kept in an excess of base, after sufficient time to establish equilibrium, the excess base is determined by titration with hydrochloric acid. Main disadvantages of this titration method are related to the presence of dissolved CO_2 that forms in the solutions after a relatively long equilibration time (Figure S5).

Procedure 1S. Estimation of the quantity of acidic groups in the graphite oxide dispersion

Acid-base titration was carried out with an automatic titrator with potentiometric determination of the titration endpoint. Using a pipette, 2.5 ml of GO and 5.0 ml of the base solution were transferred into a titration vessel, purged with argon for 5 min, the magnetic stir bar was placed, and the vessel was covered with Parafilm® Sealing Film. The vessel was placed on the magnetic stirrer and stirred at room temperature and 800 rpm for 15 min. 100 ml of deionized water was added to the titration vessel. The samples were titrated with 50 mM HCl under constant stirring.

$$c_{\mathrm{NaOH}} = \frac{V_{EP\ \mathrm{NaOH}}(\mathrm{HCl}) \cdot c_{\mathrm{HCl}}}{V_{\mathrm{NaOH}}}$$

$$n_{\mathrm{Carboxyl\ Functional\ Group}} = \frac{c_{\mathrm{NaOH}} \cdot V_{\mathrm{NaOH}} - V_{EP\ \mathrm{NaOH}+GO}(\mathrm{HCl}) \cdot c_{\mathrm{HCl}}}{m_{\mathrm{GO}\ aliq}}$$

Before purification titration reaction have been accounted as a neutralization process of free strong acid used for oxidation and –COOH surface group as a deprotonation Brønsted acid site (e.g., carboxyl, diketone, and phenol) and lactones [7].

GO–COOH + OH^- → GO–COO^- + H_2O and strong acid H^+ + OH^- → H_2O (e.g., free sulfuric acid).

After purification the most part of strong acid was eliminated by diffusion.

Table S2: Quantity of graphene oxide acidity groups estimation.

**Analyt**	**V_{EP} (HCl), mL**	**n_{CFC}, mmol/g**	**Brief comments**
NaOH	1.55	*Not applicable*	
aqGO before dialysis	1.03	2.60	Sum of –COOH of GO and H^+ from strong acids used for chemical oxidation
aqGO after dialysis	1.37	**0.90**	Genuine acid groups of graphene surface (–COOH)

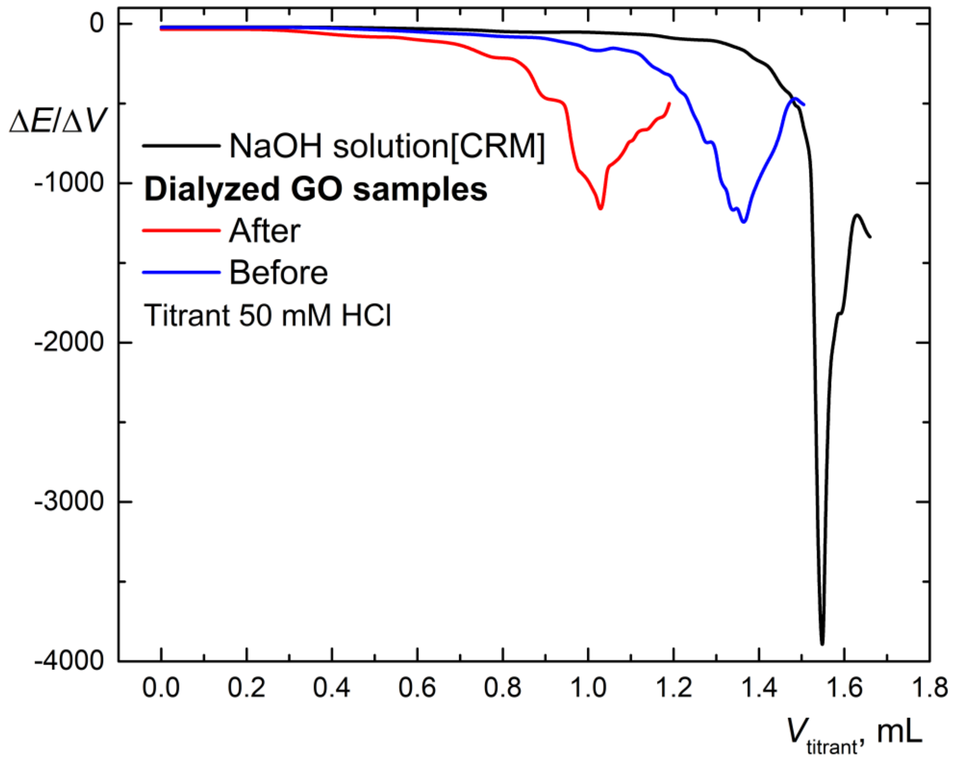

Figure S5: The Derivative End Point Methods for GO samples titration. All dependencies have shown without smoothing. (Black solid line) Diluted, Certified Standard Solutions (CRM) of NaOH; (Red solid line) GO after dialysis, (Blue solid line) GO before dialysis, which have been used to acidity group content estimation.

1.2 Finding graphene oxide surface moieties by X-ray Photoelectron Spectroscopy (XPS)

Determination of the nature of functional groups (FG) for the layers of commercial graphite oxide samples was performed by XPS (X-ray Photoelectron Spectroscopy). The results of quantitative analysis of the investigated samples are given below. To simplify processing of XPS spectra, signals from minor elements (K, N, S, Si, etc.) were not registered, therefore for all samples the total sum of contents in atomic % (integral area of a spectrum) for C and O was taken as equal to 100 %. Components in spectra of carbon for all samples we accept:

- 285 eV: –C–C– or C–H;
- ~286.5 eV: –C–O– (hydroxy-, epoxy-);
- ~288 eV: –O–C–O– or >C=O (aldo-, keto- groups);
- ~289.4 eV: >C=O (–COOH);
- from 290.5 to 291.0 eV CO_3^{2-},

but then due to the low intensity the contribution of this group can be neglected [8]. It is conventionally assumed that the C–O bond increases the bond energy in the carbon spectrum by approximately 1.5 eV. The deconvolution of the C–C band into *sp*³–C and *sp*²–C was neglected because of insufficient spectral resolution [9] (see Figure S6).

Table S3: C/O ratio estimation by different methods.

**Badge name**	**C, at.% by XPS**	**C:O ratio**	**C, at.% by supplier**	**C:O ratio**	**C, at.% by supplier corrected on anions**	**C:O ratio**	**C:O ratio by gravimetry**
GO pristine sample by modified Hummer's procedure	65.1	1.90	46.0	1.25	46.0	1.80	1.96

According to existing data, the ratio of C:O atoms for the classical methods of graphite oxide production can vary in a wide range due to the number of consecutive stages of oxidation by the methods: Brody 2.4÷3.3 [10], Staudenmaier 2.2÷3.0 [11], Hoffmann 2.5÷3.0 [12], Hammers (Hoffmann) 2.2÷2.4 [13], Hammers modified method proposed in [14] to obtain highly oxidized graphite oxide with C:O atom ratio <2.

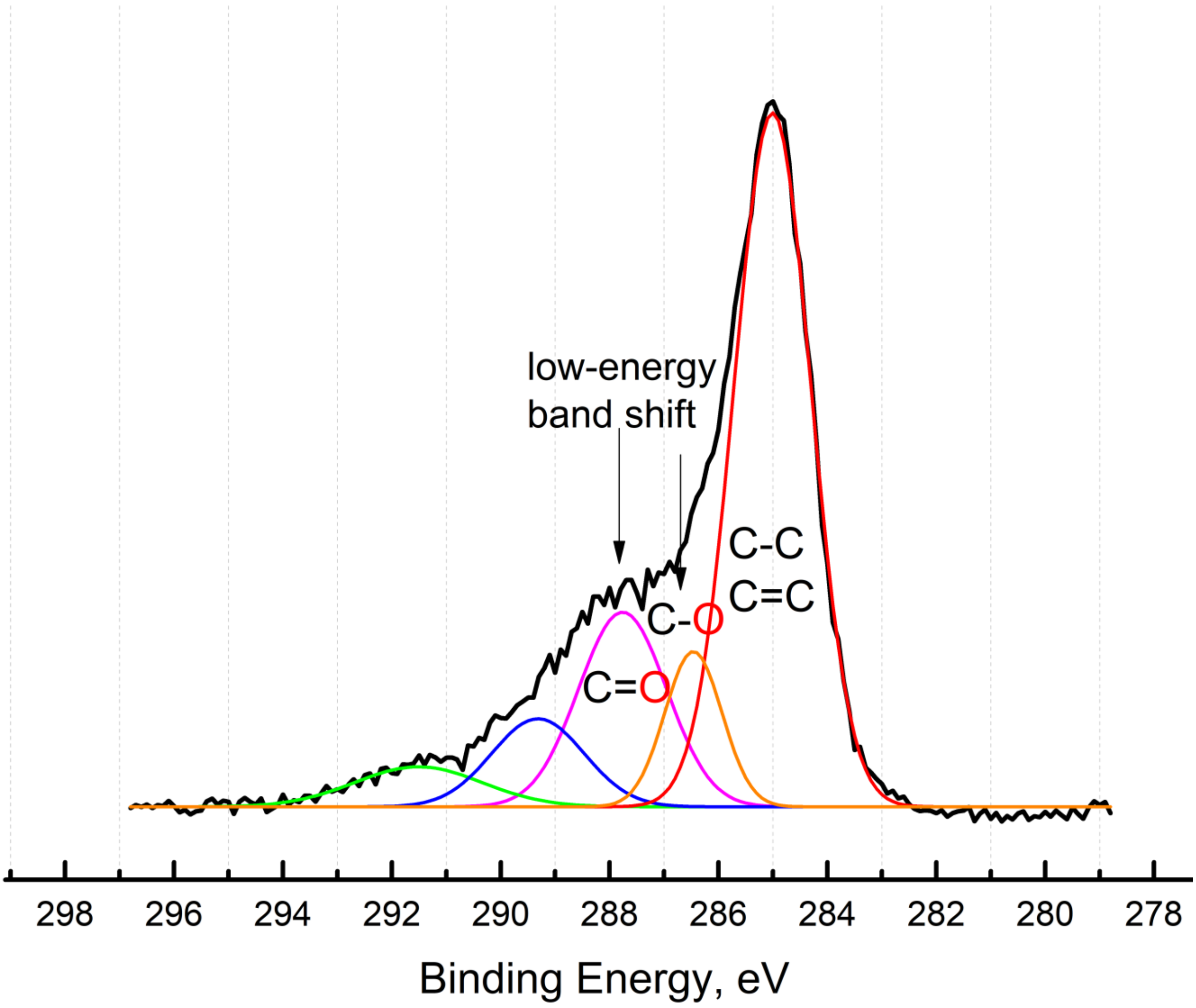

Figure S6: XPS spectra of bulk graphene oxide sample, which was used in this work.

1.3 XPS accuracy estimation by gravimetry

It is known that thermal properties of graphene depend on many parameters, such as particle size, the number of layers, defects, and the presence of oxygen functional groups (such as –COOH, –OH, C–O–C, >C=O, etc.).

1. When the OH sample is heated to 105°C, the first point of mass loss is detected. This is due to evaporation of water enclosed in the pores of the samples [15].

2. Further significant mass loss is noticed when passing the temperature range from 105 to 200°C. This mass loss point is explained by the complete decarboxylation of graphite and the loss of part of the –ON groups after heat treatment, which are transformed into a mixture of CO and CO_2 [16]. In addition, another part of the –ON is eliminated with subsequent transformation into C–O–C groups [17].

3. When reaching the point of 600°C, the third step of mass loss for graphite oxide occurs, which is connected with destruction of C–O–C bonds and the following oxidation, then transition of these groups into CO and CO_2 that is confirmed by IR spectroscopy with annealing at 350°C where the presence of emitting gases was also confirmed by gas detectors [18].

The mass loss at temperatures up to 200°C does not depend on the lateral size of the graphite particles and the number of graphene layers, but during the calcination at temperatures >360°C this dependence is observed, so it was found that with increasing particle size the maximum temperature of complete combustion of –OH increases [15].

Procedure 2S. Estimation of the masses of the functional composition of graphite oxide and C/O ratio.

A porcelain crucible is brought to a constant weight; for this purpose, it is ignited at 800°C for 30 min, after which it is cooled in a desiccator and weighed. Losses of weight on ignition are studied at 105, 200, 600, 800°C, hardening isothermal for 20, 30, 30, and 30 min, respectively. Next, the sample is cooled down in a desiccator and weighed. Temperature heating to a predetermined temperature during the whole experiment is carried out according to the program, 3°C/min. Then, the mass fraction and mass loss on ignition is calculated.

1.4 Pre-soaking regent's tanks. Estimation of plasticizer quantity.

The residual content of phthalates was checked by gas chromatography-mass spectrometry. Preliminary, liquid-liquid extraction with *n*-hexane was carried out. The extract was dried off by anhydrous and calcined sodium sulfate and after injected into the injection port of the chromatograph. The reagents that were in the container for 60 days were analyzed: water, hydrogen peroxide, and EDTA.

Analysis conditions:

An Agilent 8890 GC gas chromatograph equipped with an MPS Gerstel automatic sampler and an Agilent 5977B Inert Plus MSD mass spectrometer (Agilent Technologies, USA) were used for residual plasticizer quantity determination. Data collection and chromatogram processing were performed using MassHunter software (Agilent Technologies, USA). Capillary column HP-5MS (30 m×0.25 mm, 0.25 μm) with a stationary phase based on methyl (95%)-phenyl (5%)-polysiloxane. The carrier gas is high-pure (99.999%) helium, the carrier gas flow through the column is 1 cm^3/min. The volume of the injected sample is 1 mm^3 (1 μL). Splitless sample injection. Evaporator temperature 280°C, interface temperature 290°C; temperature gradient to separate the components.

Temperature program: initial temperature of the column 50°C, isotherm for 4 minutes, with a rise rate of 30°C/min to 280 °C, then with a rise rate of 5°C/min to 310°C isotherm at 310°C for 2 min.

The energy of ionizing electrons is 70 eV, the temperature of the quadrupole is 150°C, and that of the ion source is 230°C. Registration of chromatograms in scanning mode m/z from 29 to 400.

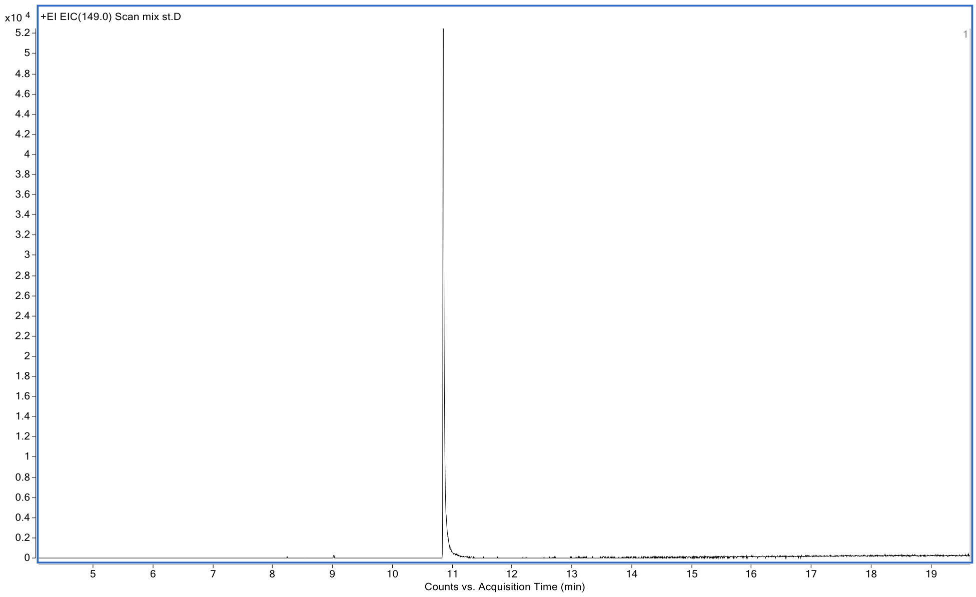

Figure S7: Dibutyl phthalate (DBP) 1.6 ppm SRM in *n*-hexane (n=3, P=0.95)

Table S4: Phthalate estimation by GC-MS.

**Sample badge (canister of regent)**	**Dibutyl phthalate (DBP), ppb**	**Diisobutyl phthalate (DIBP), ppb**	**Diethylhexyl phthalate (DEHP), ppb**
**t_r, min**	**11.85**	**11.53**	**12.72**
H_2O_2	<0.1	<0.1	<0.1
H_2O	<0.1	<0.1	<0.1
EDTA	1.6 ± 0.05	1.3 ± 0.03	<0.1

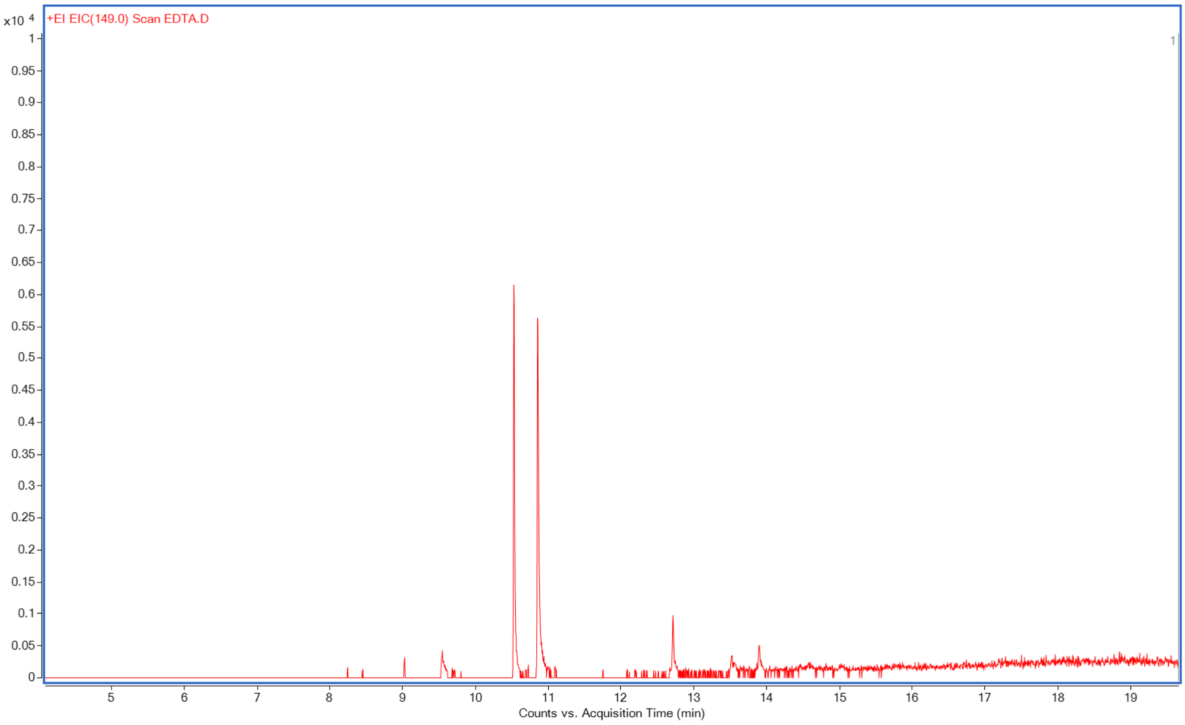

Figure S8: Typical chromatogram from EDTA solution. The DBP, DIBP, and DEHP are observed.

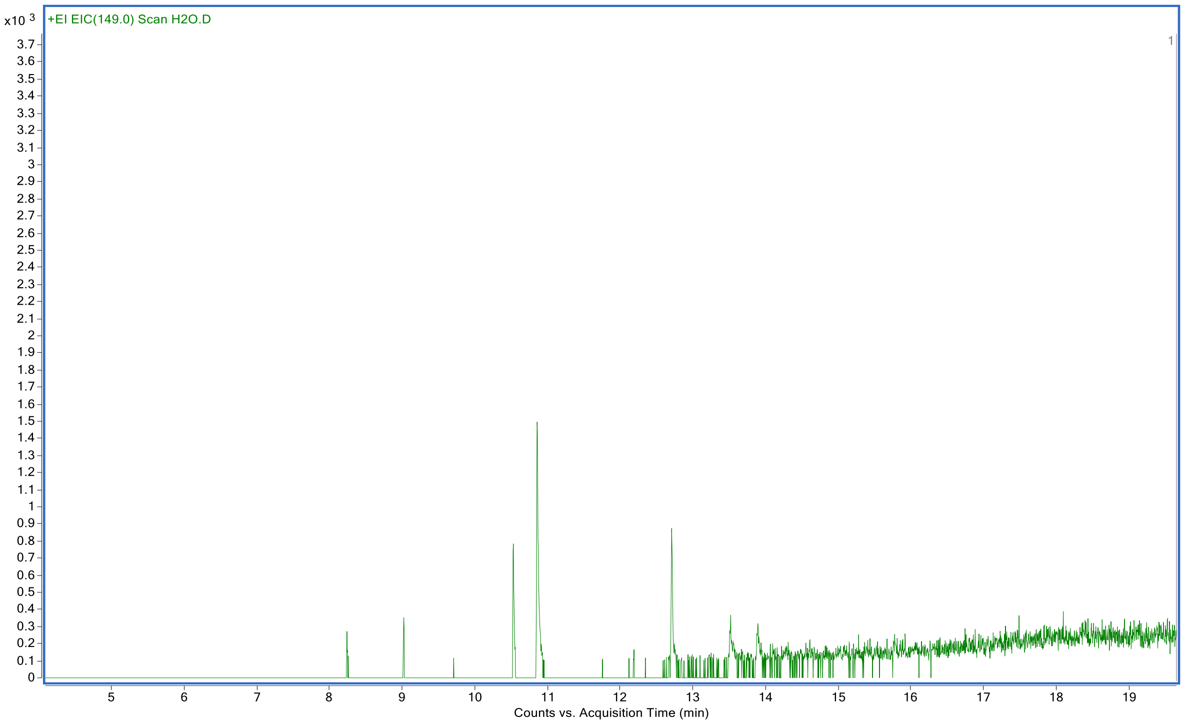

Figure S9: Typical chromatogram of H_2O_2 solution. The DBP, DIBP, and DEHP are not observed (same as blank solution).

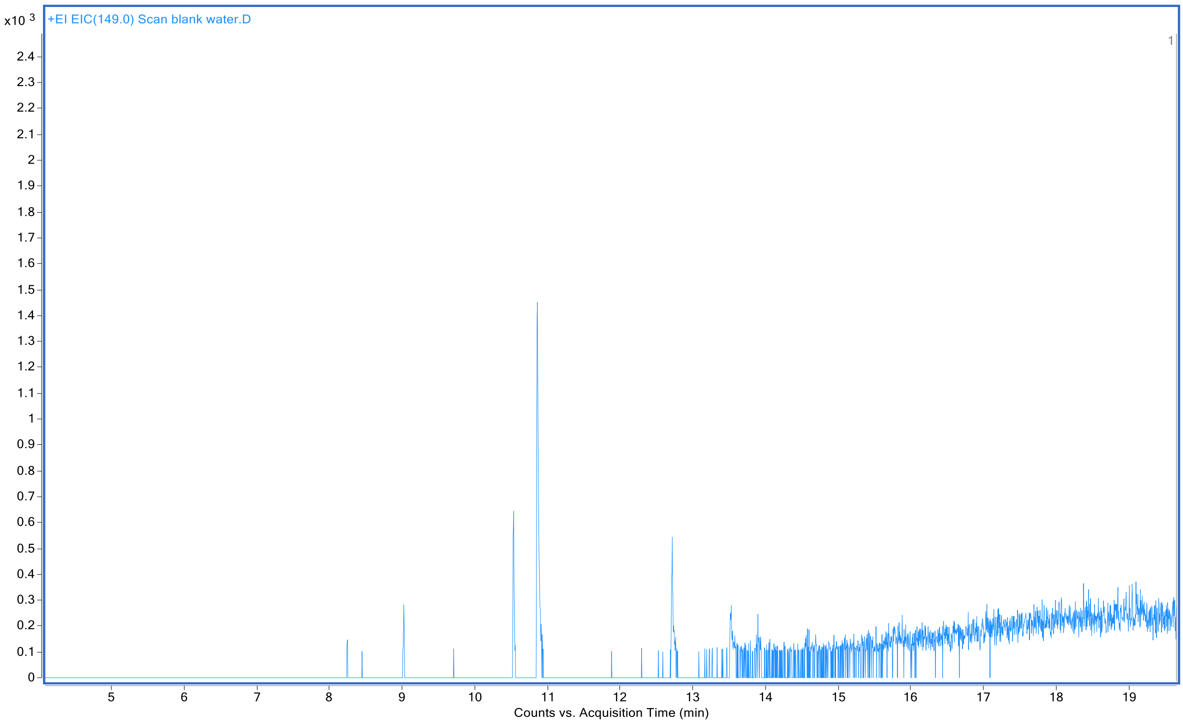

Figure S10: Typical chromatogram of H_2O solution. The DBP, DIBP, and DEHP are not observed (same as blank solution).

Thus, after 60 days of storage reagents at room temperature we observed, that plasticizer do not release in H_2O and H_2O_2 tanks, but EDTA solution have less 1 ppm of dibutyl phthalate and diisobutyl phthalate.

1.5 Raman spectra for GO sample characterisation

Raman spectroscopy was measured using Confotech NR-500 spectrometer (SOL Instruments) with 633 nm laser using x40 (NA=0.75) lens at ~3 mW laser power.

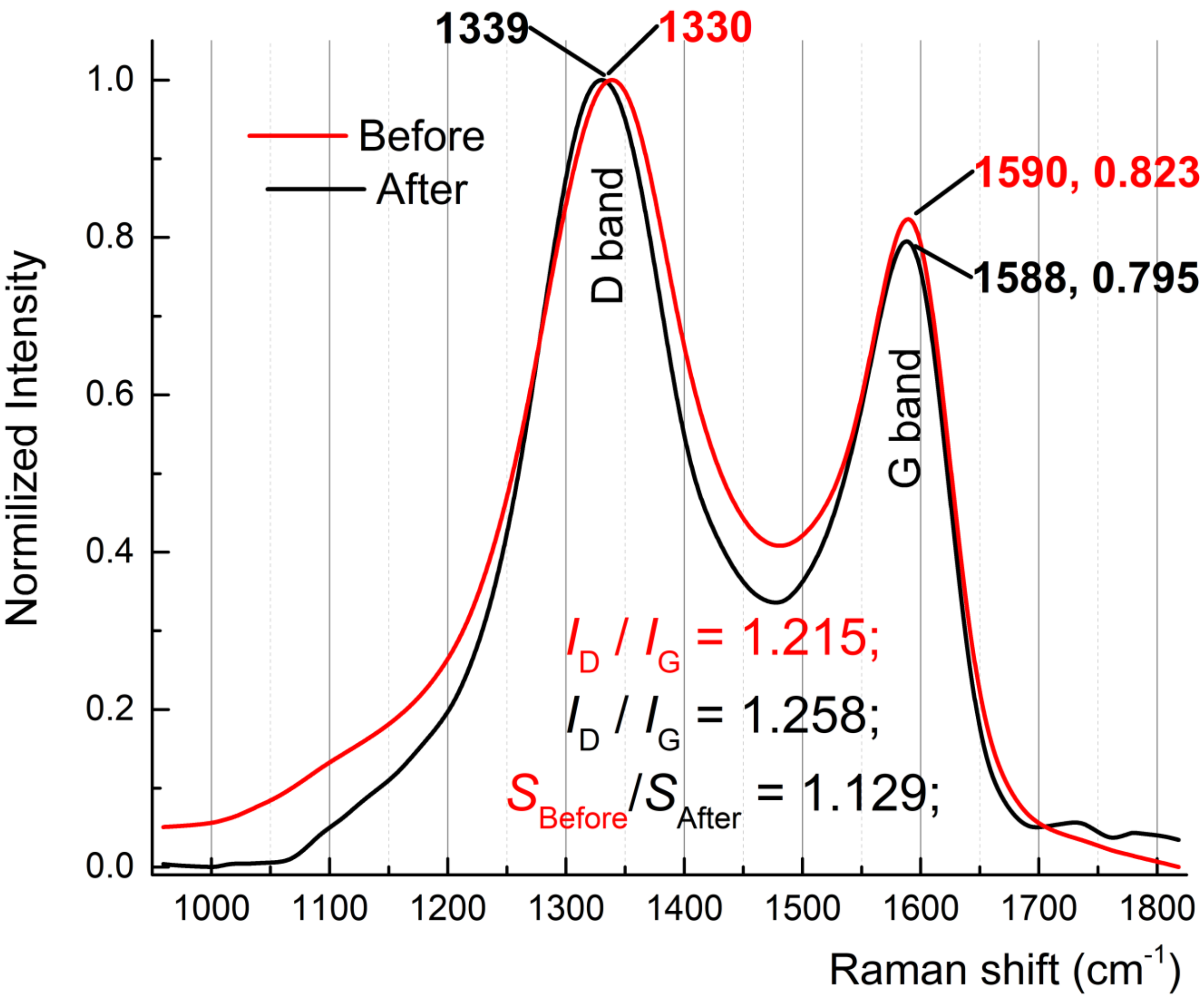

Figure S11: Typical normalized Raman spectra for GO samples (black solid line) after dialysis, (red solid line) before dialysis.

The results showed that the Raman spectra of these materials presented D and G bands centered at about 1330 and 1590 cm^{-1}, respectively (Figure S.11). Raman spectroscopy confirmed the GO structure preservation during the purification process: samples had both G and D vibration bonds of sp^2 carbon. Bands intensity ratio (I_D/I_G) has the same order of magnitude, but after dialysis process, it increased by 3.5%, which indicated the better alignment of carbon atoms structure to perfect hexagonal honeycomb, which resulted from a less structured distortion induced by coordination of GO functional groups with more labile water molecules instead of strongly bonding metal ions removed during the purification process. The increase of sp^2 hybridization and monolayer content favors the

increase of the free-radical scavenging activity of purified graphene [19]. In addition, the total area under the curve decreased after the purification process that proves changes in samples. Crystallinity structure band D has shifted from 1330 to 1339 cm^{-1} (Δ=9 cm^{-1}).

Supplementary References List

1. Guo, S.; Zhang, G.; Guo, Y.; Yu, J.C. Graphene oxide–Fe2O3 hybrid material as highly efficient heterogeneous catalyst for degradation of organic contaminants. *Carbon* **2013**, *60*, 437-444, doi:https://doi.org/10.1016/j.carbon.2013.04.058.
2. Matsumura, Y.; Hagiwara, S.; Takahashi, H. Automatic potentiometric titration of surface acidity of carbon black. *Carbon* **1976**, *14*, 163-167, doi:https://doi.org/10.1016/0008-6223(76)90097-X.
3. Sun, C.; Berg, J.C. A review of the different techniques for solid surface acid-base characterization. *Advances in colloid and interface science* **2003**, *105*, 151-175, doi:10.1016/s0001-8686(03)00066-6.
4. Langley, L.A.; Villanueva; E., D.; Fairbrother, D.H. Quantification of Surface Oxides on Carbonaceous Materials. *Chemistry of Materials* **2006**, *18*, 169-178.
5. Szabó, T.; Tombácz, E.; Illés, E.; Dekany, I. Enhanced acidity and pH-dependent surface charge characterization of successively oxidized graphite oxides. *Carbon* **2004**, *44*, 537-545, doi:10.1016/j.carbon.2005.08.005.
6. Long, C.M.; Nascarella, M.A.; Valberg, P.A. Carbon black vs. black carbon and other airborne materials containing elemental carbon: Physical and chemical distinctions. *Environmental Pollution* **2013**, *181*, 271-286, doi:https://doi.org/10.1016/j.envpol.2013.06.009.
7. Zhang, Z.; Flaherty, D.W. Modified potentiometric titration method to distinguish and quantify oxygenated functional groups on carbon materials by pKa and chemical reactivity. *Carbon* **2020**, *166*, 436-445, doi:https://doi.org/10.1016/j.carbon.2020.05.040.
8. Linstorm, P. Nist chemistry webbook, nist standard reference database number 69. *J. Phys. Chem. Ref. Data, Monograph* **1998**, *9*, 1-1951.
9. Park, S.; An, J.; Jung, I.; Piner, R.D.; An, S.J.; Li, X.; Velamakanni, A.; Ruoff, R.S. Colloidal Suspensions of Highly Reduced Graphene Oxide in a Wide Variety of Organic Solvents. *Nano Letters* **2009**, *9*, 1593-1597, doi:10.1021/nl803798y.
10. Brodie, B.C. XIII. On the atomic weight of graphite. *Philosophical Transactions of the Royal Society of London* **1859**, *149*, 249-259, doi:10.1098/rstl.1859.0013.
11. Staudenmaier, L. Verfahren zur Darstellung der Graphitsäure. *Berichte der deutschen chemischen Gesellschaft* **1898**, *31*, 1481-1487, doi:https://doi.org/10.1002/cber.18980310237.
12. Hofmann, U.; König, E. Untersuchungen über Graphitoxyd. *Zeitschrift für anorganische und allgemeine Chemie* **1937**, *234*, 311-336, doi:https://doi.org/10.1002/zaac.19372340405.
13. Hummers, W.S.; Offeman, R.E. Preparation of Graphitic Oxide. *Journal of the American Chemical Society* **1958**, *80*, 1339-1339, doi:10.1021/ja01539a017.
14. Marcano, D.C.; Kosynkin, D.V.; Berlin, J.M.; Sinitskii, A.; Sun, Z.; Slesarev, A.; Alemany, L.B.; Lu, W.; Tour, J.M. Improved Synthesis of Graphene Oxide. *ACS Nano* **2010**, *4*, 4806-4814, doi:10.1021/nn1006368.

15. Farivar, F.; Lay Yap, P.; Karunagaran, R.U.; Losic, D. Thermogravimetric Analysis (TGA) of Graphene Materials: Effect of Particle Size of Graphene, Graphene Oxide and Graphite on Thermal Parameters. *C* **2021**, *7*, 41.
16. Abdolhosseinzadeh, S.; Asgharzadeh, H.; Seop Kim, H. Fast and fully-scalable synthesis of reduced graphene oxide. *Scientific Reports* **2015**, *5*, 10160, doi:10.1038/srep10160.
17. Shao, G.; Lu, Y.; Wu, F.; Yang, C.; Zeng, F.; Wu, Q. Graphene oxide: the mechanisms of oxidation and exfoliation. *Journal of Materials Science* **2012**, *47*, 4400-4409, doi:10.1007/s10853-012-6294-5.
18. Sengupta, I.; Sharat Kumar, S.S.S.; Pal, S.K.; Chakraborty, S. Characterization of structural transformation of graphene oxide to reduced graphene oxide during thermal annealing. *Journal of Materials Research* **2020**, *35*, 1197-1204, doi:10.1557/jmr.2020.55.
19. Liu, Y.; Zhang, S.; Pei, X.; Shi, H.; Li, D.; Xu, Z.; Li, S.; Xue, Y.; Song, L. Free radical scavenging behavior of multidimensional nanomaterials in γ-irradiated epoxy resin and mechanical and thermal performance of γ-irradiated composites. *Composites Part C: Open Access* **2021**, *4*, 100095, doi:https://doi.org/10.1016/j.jcomc.2020.100095.
